# Genetic Variants Associated with Serum Thyroid Stimulating Hormone (TSH) Levels in European Americans and African Americans from the eMERGE Network

**DOI:** 10.1371/journal.pone.0111301

**Published:** 2014-12-01

**Authors:** Jennifer R. Malinowski, Joshua C. Denny, Suzette J. Bielinski, Melissa A. Basford, Yuki Bradford, Peggy L. Peissig, David Carrell, David R. Crosslin, Jyotishman Pathak, Luke Rasmussen, Jennifer Pacheco, Abel Kho, Katherine M. Newton, Rongling Li, Iftikhar J. Kullo, Christopher G. Chute, Rex L. Chisholm, Gail P. Jarvik, Eric B. Larson, Catherine A. McCarty, Daniel R. Masys, Dan M. Roden, Mariza de Andrade, Marylyn D. Ritchie, Dana C. Crawford

**Affiliations:** 1 Department of Biomedical Informatics, Vanderbilt University, Nashville, TN, United States of America; 2 Department of Medicine, Vanderbilt University, Nashville, TN, United States of America; 3 Department of Molecular Physiology and Biophysics, Vanderbilt University, Nashville, TN, United States of America; 4 Center for Human Genetics Research, Vanderbilt University, Nashville, TN, United States of America; 5 Division of Epidemiology, Department of Health Sciences Research, Mayo Clinic, Rochester, MN, United States of America; 6 Office of Research, Vanderbilt University, Nashville, TN, United States of America; 7 Division of Biomedical Statistics and Informatics, Department of Health Sciences Research, Mayo Clinic, Rochester, MN, United States of America; 8 Biomedical Informatics Research Center, Marshfield Clinic Research Foundation, Marshfield, WI, United States of America; 9 Group Health Research Institute, Seattle, WA, United States of America; 10 Department of Medicine, Northwestern University, Chicago, IL, United States of America; 11 Division of Genomic Medicine, National Human Genome Research Institute, Bethesda, MD, United States of America; 12 Division of Cardiovascular Diseases, Department of Health Sciences Research, Mayo Clinic, Rochester, MN, United States of America; 13 Center for Genetic Medicine, Northwestern University, Chicago, IL, United States of America; 14 Essentia Institute of Rural Health, Duluth, MN, United States of America; 15 Department of Pharmacology, Vanderbilt University, Nashville, TN, United States of America; 16 Departments of Medicine (Medical Genetics) and Genome Sciences, University of Washington, Seattle, WA, United States of America; National Cancer Institute, National Institutes of Health, United States of America

## Abstract

Thyroid stimulating hormone (TSH) hormone levels are normally tightly regulated within an individual; thus, relatively small variations may indicate thyroid disease. Genome-wide association studies (GWAS) have identified variants in *PDE8B* and *FOXE1* that are associated with TSH levels. However, prior studies lacked racial/ethnic diversity, limiting the generalization of these findings to individuals of non-European ethnicities. The Electronic Medical Records and Genomics (eMERGE) Network is a collaboration across institutions with biobanks linked to electronic medical records (EMRs). The eMERGE Network uses EMR-derived phenotypes to perform GWAS in diverse populations for a variety of phenotypes. In this report, we identified serum TSH levels from 4,501 European American and 351 African American euthyroid individuals in the eMERGE Network with existing GWAS data. Tests of association were performed using linear regression and adjusted for age, sex, body mass index (BMI), and principal components, assuming an additive genetic model. Our results replicate the known association of *PDE8B* with serum TSH levels in European Americans (rs2046045 p = 1.85×10^−17^, β = 0.09). *FOXE1* variants, associated with hypothyroidism, were not genome-wide significant (rs10759944: p = 1.08×10^−6^, β = −0.05). No SNPs reached genome-wide significance in African Americans. However, multiple known associations with TSH levels in European ancestry were nominally significant in African Americans, including *PDE8B* (rs2046045 p = 0.03, β = −0.09), *VEGFA* (rs11755845 p = 0.01, β = −0.13), and *NFIA* (rs334699 p = 1.50×10^−3^, β = −0.17). We found little evidence that SNPs previously associated with other thyroid-related disorders were associated with serum TSH levels in this study. These results support the previously reported association between *PDE8B* and serum TSH levels in European Americans and emphasize the need for additional genetic studies in more diverse populations.

## Introduction

Hyperthyroidism and hypothyroidism are important endocrine diseases caused by over- or under-production of thyroid hormone, which is regulated by thyroid stimulating hormone (TSH) produced in the anterior pituitary gland. Hypothyroidism, the most common thyroid disease, can be caused by iodine insufficiency, autoimmunity, pregnancy, pituitary disease (leading to increased TSH production), or other conditions. Thyroid diseases occur more often in women than in men [1] and the risk of developing hypothyroidism increases with age [2], [3]. Diagnosis of thyroid diseases involves measuring TSH levels and circulating thyroxine (T4) and triiodothyronine (T3) in the blood; elevated TSH levels and depressed T4 levels signify clinical hypothyroidism [2],[4] while elevated TSH levels and normal T4 levels indicate mild (subclinical) hypothyroidism [5]. TSH is produced by a normally functioning pituitary gland in response to decreased thyroid hormone levels; as thyroid hormone levels decrease, TSH signals to the thyroid to produce additional thyroid hormone. When the thyroid gland does not maintain sufficient production of thyroid hormone, serum TSH levels become elevated, and the individual develops hypothyroidism. Similarly, elevated thyroid hormone levels from primary hyperthyroidism result in decreased TSH levels.

Both genetic and environmental factors influence serum TSH levels. Neonatal TSH levels have been associated with maternal characteristics such as nulliparity, preeclampsia, and induced labor [6]. Among adults, physical and emotional stress, poor nutrition, increased body mass index (BMI), smoking, and pregnancy are all risk factors for elevated serum TSH levels [7]–[9]. Normal serum TSH levels range from 0.3 µIU/mL–4.0 µIU/mL but are tightly regulated within an individual, suggesting a genetic ‘set point’ for individual thyroid hormone levels [5], [10], [11]. A cross-sectional population study demonstrated differences in mean TSH levels between race/ethnicities, with higher mean TSH levels in non-Hispanic whites than in Mexican Americans or non-Hispanic blacks [5]. The etiology behind the observed differences in mean TSH levels across ethnic groups has not been elucidated, and it is unclear if those differences lead to lower prevalence of hypothyroidism in populations of diverse ancestry. A recent study identified differences in prevalence of thyroid cancer across ethnic groups living in England [12], and TSH antibodies were demonstrably lower in non-Hispanic blacks compared to non-Hispanic whites or Mexican-Americans in the National Health and Nutrition Examination Survey (NHANES) III [13]; however, studies evaluating hypothyroidism or hyperthyroidism burden among different racial/ethnic groups have not been performed. Twin and family-based studies have suggested heritability estimates of 32%-67% for TSH, T4, and T3 levels [14]–[16], and a recent study found heritability for TSH to be 58% in newborn twins[17]. These data taken together suggest TSH level variation is largely a product of genetic factors, corroborating the hypothesis that each individual maintains a set-point for TSH levels. Several genetic association studies have been performed, including two meta-analyses of GWAS [18], [19]. These studies have identified common variants associated with serum TSH levels: rs2046045 (*PDE8B*), rs10917477 (*CAPZB*), rs10028213 (*NR3C2*), and rs3813582 (16q23) [18],[20]. Altogether, the known loci explain <5% of the variance in TSH levels [19]. However, these GWAS and meta-analyses have been performed in populations of European ancestry, and it is unclear if these findings generalize to other race/ethnicities.

In this study, we sought to identify variants associated with normal variability of serum TSH levels in euthyroid (thyroid disease free) European Americans and African Americans from the Electronic Medical Records and Genomics (eMERGE) Network. We looked to replicate in our study known associations between SNPs and serum TSH levels. We hypothesized variants associated with serum TSH levels might also be associated with thyroid disorders, such as hyperthyroidism (Grave's disease), hypothyroidism (Hashimoto's disease), and thyroid cancer. Given that increased BMI is a risk factor for elevated serum TSH levels, we also tested for evidence that TSH-associated SNPs are modified by BMI in this study of euthyroid European and African Americans from the eMERGE Network.

## Methods

### eMERGE

The eMERGE Network is a collaboration of institutions with biobanks linked to EMRs. The data for these analyses included Phase I of the eMERGE Network whose members included Group Health Cooperative/University of Washington, Marshfield Clinic, Mayo Clinic, Northwestern University, Vanderbilt University and the eMERGE Administrative Coordinating Center [21].

### Study Population

This study was performed in the eMERGE Network which includes approximately 17,000 individuals who were phenotyped and genotyped for previous studies investigating a variety of complex diseases (e.g. dementia, cataracts, peripheral arterial disease (PAD), type 2 diabetes) and medically relevant quantitative traits (e.g. cardiac conduction) [22]. To qualify for euthyroid designation in this analysis, individuals were required to have at least one test of thyroid function (i.e., TSH and T3 or T4 if available) with no abnormal results, must not have any billing codes for hypothyroidism or history of myasthenia gravis in his/her EMR or evidence of thyroid replacement medication, and must have at least two past medical history sections (non-acute visits) and medication lists. For individuals with multiple TSH tests, the median TSH level was used in the analysis. Individuals were excluded if they had any cause of hypothyroidism or hyperthyroidism, any other thyroid diseases (e.g. Graves, thyroid cancer) as indicated by billing (ICD-9) codes, procedure (CPT) codes or text word diagnoses, or were on thyroid-altering medication (e.g., lithium) [22]. From this group, 6,086 European Americans and 633 African Americans qualified as euthyroid, of which 4,501 European Americans and 351 African Americans had body mass index (BMI). The appropriate institutional review board at each participating study site approved all procedures.

### Genotyping

Genotyping was performed using the Illumina Human660W-Quadv1_A and the Illumina1M BeadChips for European Americans and African Americans, respectively, as previously described [22]. Of the SNPs on each array, 474,366 SNPs and 905,285 SNPs, respectively, passed quality control filters for tests of genotyping efficiency (>99% call rate), and minor allele frequency (>5%). Details of eMERGE quality control have been previously published [23],[24]. eMERGE Network data have been deposited into the Database for Genotypes and Phenotypes (dbGaP).

### Statistical Analysis

Quality control and data analysis were performed using a combination of PLINK [25],[26], and R software, and data were plotted using R code obtained from the Getting Genetics Done website [27],[28], Stata [29] and Synthesis-View [30]. Power calculations were performed using Quanto [31]. Linear regression was performed assuming an additive genetic model to test for associations between individual SNPs and log-transformed median serum TSH levels. Tests were performed stratified by race/ethnicity, unadjusted and adjusted for age, sex, BMI, and first principal component (PC1) calculated with EIGENSTRAT [32]. Control for population stratification was evaluated with Q-Q plots and calculation of the lambda statistic using R packages qqman and GenABEL [33]. No evidence of residual population stratification was observed in the European Americans (λ = 1.04) or African Americans (λ = 1.00). Additional tests of association were performed in European Americans stratified by BMI (normal: BMI 18.5–24.9; overweight: BMI ≥25 and normal; overweight: BMI ≥25–30; obese: BMI >30) and adjusted for age, sex, and PC1. We also performed formal tests of interaction between SNPs associated with TSH levels as a significance threshold of p<1×10^−04^ and stratified BMI (normal versus overweight) stratified by race/ethnicity in adjusted (age, sex, PC1, and main effects) models. We considered a SNP-BMI interaction significant at a threshold of p<0.05. Wilcoxon rank-sum tests were performed to compare median TSH levels at each genotype for normal vs. overweight BMI categories for each SNP for the normal/overweight BMI analysis and Bonferroni-corrected multiple pairwise analysis following ANOVA for the normal/overweight/obese BMI analysis.

In addition to GWAS discovery, we sought to replicate and generalize previously reported genetic associations for TSH levels. We considered a SNP replicated in European Americans if the tested SNP was identical to the index SNP, or a proxy in strong linkage disequilibrium (LD) (r^2^>0.7) with the index SNP in 1000 Genomes CEU reference panel, and the direction of effect was consistent with the previous report after taking into account coding allele differences. We considered a SNP generalized to African Americans if the tested SNP was identical to, or a proxy in strong LD with (r^2^>0.7), the index SNP in 1000 Genomes CEU reference panel, and the direction of effect was consistent with European Americans. For the replication/generalization analysis, significance was defined at a threshold of p<0.05. Power calculations were performed assuming the genetic effect sizes reported in the literature, the present study sample size, and the present study coded allele frequencies.

## Results

### Study participants

All eMERGE participating sites contributed data for European Americans and all sites except Marshfield Clinic contributed data for African Americans (Table S1). Collectively, European Americans had higher mean TSH levels compared to the African Americans (1.90 µIU/mL vs. 1.45 µIU/mL), had lower BMI (27.51 kg/m^2^ vs. 32.16 kg/m^2^), included more men (52.19% male vs. 25.07%), and were older (median decade of birth 1930s vs. 1950s) (Table 1). The higher mean TSH level in European Americans compared to African Americans is consistent with previous epidemiologic reports [1],[5],[34]. The age, BMI, and sex ratio differences between the groups observed here most likely reflect ascertainment differences resulting from the characteristics of the source populations at each eMERGE site, rather than true differences at the overall population level.

**Table 1:** Population characteristics in euthyroid individuals for serum thyroid stimulating hormone (TSH) levels and demographics in the eMERGE Network.

	European Americans (n = 4,501)	African Americans (n = 351)
**Female (%)**	47.81	74.93
**Body mass index, kg/m^2^**	27.51 (5.55)	32.16 (8.43)
**Age at lab, years**	65.50 (12.48)	50.59 (18.41)
**TSH levels, µIU/mL**	1.90 (0.93)	1.45 (0.72)
**Decade of birth, (%)**		
**1910s**	608 (13.51)	30 (8.55)
**1920s**	865 (19.22)	40(11.40)
**1930s**	994 (22.08)	22 (6.27)
**1940s**	1246 (27.68)	40 (11.40)
**1950s**	612 (13.60)	71 (20.23)
**1960s**	89 (1.98)	67 (19.09)
**1970s**	48 (1.07)	42 (11.97)
**1980s**	38 (0.84)	38 (10.83)
**1990s**	1 (0.02)	1 (0.28)

Means (standard deviation) are presented unless otherwise noted.

### TSH levels: Discovery

**Figure 1:**
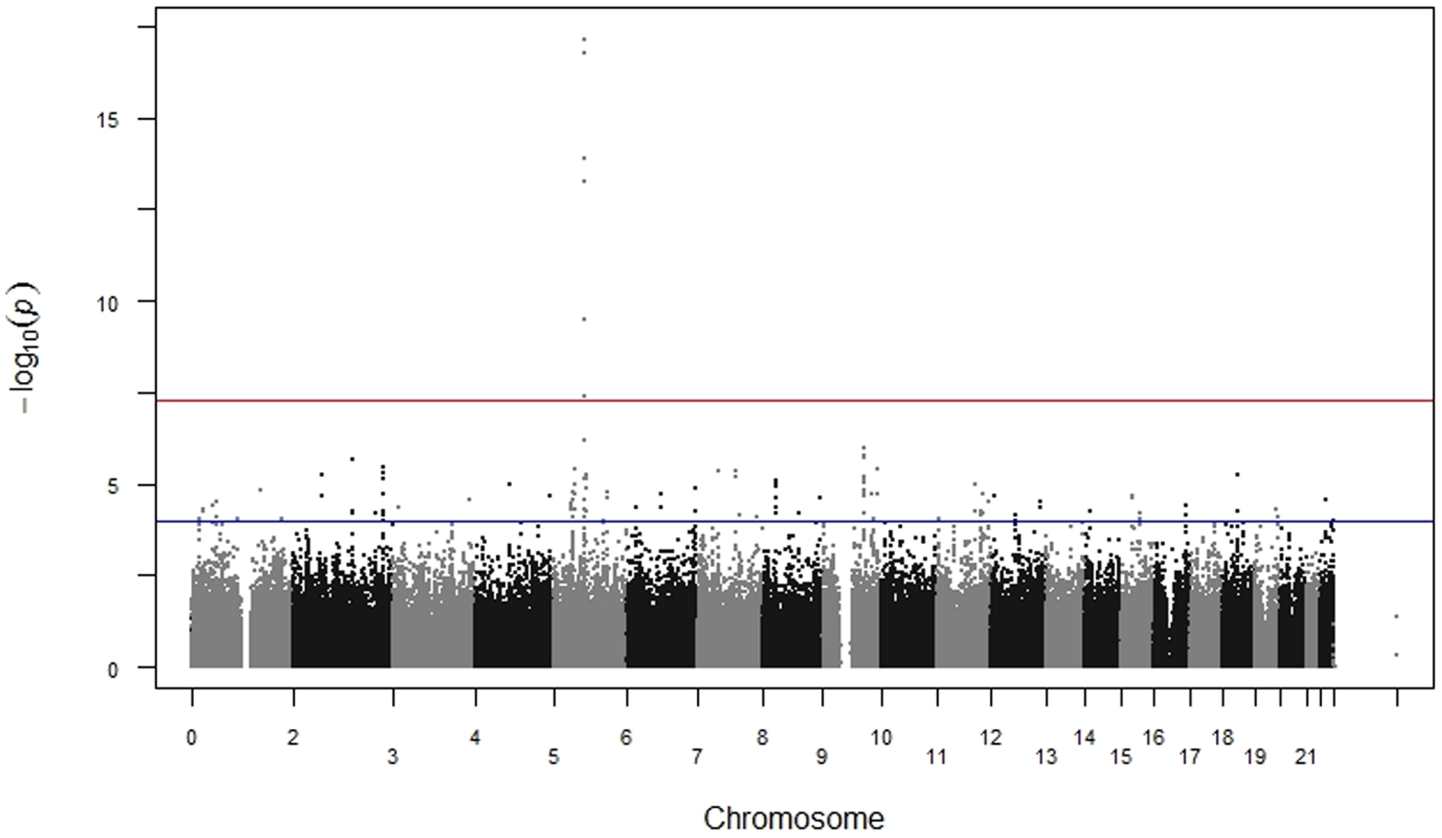
Manhattan plot of tests of association with serum thyroid stimulating hormone (TSH) levels in euthyroid European Americans in eMERGE. Data shown are p-values from single SNP tests of association with serum TSH levels in a model adjusted for age, sex, principal component (PC1), and body mass index in euthyroid European Americans in eMERGE Network (n = 4,501). The y-axis represents the –log10 (p-value); horizontal lines represent Bonferroni corrected significance level (p<5×10^−08^) (top) and suggestive significance level (1×10^−04^) (bottom). Chromosomes are arranged on the x axis.

We performed standard single SNP tests of association stratified by race/ethnicity and adjusted for sex, age (decade of birth), BMI, and PC1. For European Americans, we identified six SNPs in *PDE8B* on chromosome 5 as associated with TSH levels at genome-wide significance (Figure 1; Table 2). Our most significant result, rs1382879, was a perfect proxy for previously-identified [19] rs2046045 (r^2^ = 1.00) and was in moderate-to-high LD (r^2^>0.30) with the other significant *PDE8B* SNPs. No novel genotype-phenotype associations were identified at genome-wide significance in this sample of European Americans. However, an additional 111 SNPs were suggestively associated with serum TSH levels (p<1×10^−4^), including seven SNPs in *PDE8B*, ten SNPs near *FOXE1*, three SNPs in *PDE10A*, four SNPs in *THBS4*, and eight SNPs in *NRG1* (Table S2). The majority of these SNPs are located in noncoding regions of the genome (intronic, upstream, downstream); however, rs3745746 (*CABP5*, p = 4.93×10^−5^) is a missense mutation, and rs1443434 (*FOXE1*, p = 6.53×10^−5^) is located in the 3′ untranslated region.

**Table 2:** Genome-wide significant SNP associations for serum thyroid stimulating hormone (TSH) levels in eMERGE euthyroid European Americans (n = 4,501).

CHR	SNP	GENE	GENE REGION	CODED ALLELE	BETA (SE)	P-VALUE
5	rs1382879	*PDE8B*	intronic	G	0.09 (0.01)	7.16×10^−18^
5	rs2046045	*PDE8B*	intronic	C	0.09 (0.01)	1.85×10^−17^
5	rs989758	*PDE8B*	intronic	T	0.08 (0.01)	1.33×10^−14^
5	rs9687206	*PDE8B*	intronic	G	0.08 (0.01)	5.52×10^−14^
5	rs12515498	*PDE8B*	intronic	C	0.07 (0.01)	3.27×10^−10^
5	rs6885813	*PDE8B*	intronic	A	0.06 (0.01)	4.05×10^−08^

Significance defined as p<5×10^−8^. Tests of association using linear regression for 474,366 SNPs assuming an additive genetic model and adjusted for age, sex, principal component (PC1), and body mass index were performed.

No SNPs were associated with TSH levels in African Americans at the genome-wide significance threshold of p<5.0×10^−8^ (Figure S1). However, 87 SNPs reached a suggestive significance level (p<1×10^−4^); the most significant result was rs1409005 (*POU4F1-AS1*, p = 5.02×10^−7^). Similar to the results in the European Americans, the majority of these SNPs were located in noncoding regions except for two missense mutations (*COQ5* rs3742049, p = 6.08×10^−5^; *RBM20* rs942077, p = 8.47×10^−5^) and one synonymous substitution (*KLK1* rs1054713, p = 4.16×10^−5^) (Table S3).

### Trans-population genetic associations

**Figure 2:**
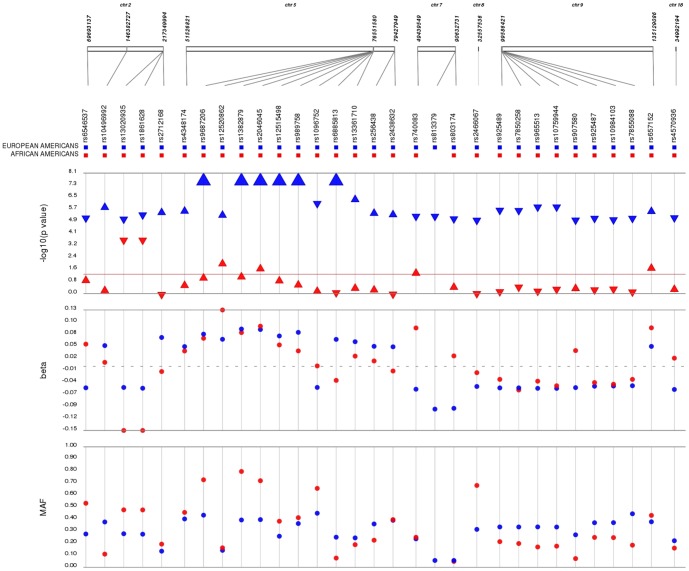
Comparison of most significant associations identified in European Americans with African Americans from the eMERGE Network. We plotted p-values, coded allele frequencies, and betas for euthyroid European Americans (n = 4,501) and African Americans (n = 351) in the eMERGE Network for serum TSH level tests of association using SynthesisView. Data shown are comparisons between European Americans (blue markers) and African Americans (red markers) for p-values (data shown are –log10 (pvalue)), genetic effect magnitudes (beta), and minor (coded) allele frequencies (MAF) for the 31 most significant SNPs in European Americans. Red horizontal line on p-value track indicates p = 0.05. SNPs are oriented across the top of the figure, arranged by chromosomal location. Large triangles represent p-values at or smaller than 5×10^−08^. Direction of the marker for p-values indicates direction of effect for each SNP.

Given the smaller sample size of African Americans with serum TSH levels, the GWAS was underpowered to detect associations at genome-wide significance with expected small to moderate effect sizes. Therefore, we evaluated the 31 most significant (p<1×10^−5^) associations from the European American dataset for evidence of generalization to the African American dataset at a liberal significance threshold of 0.05 (Figure 2). One SNP, rs813379, was not directly genotyped in African Americans. We observed two SNPs in *PDE8B* associated with serum TSH levels in European Americans (rs2046045: p = 1.85×10^−17^ and rs12520862: p = 7.48×10^−6^) that were also associated in African Americans (p = 0.03 and 0.01, respectively) with consistent directions and magnitude of effect after accounting for the coded allele. We also observed two SNPs upstream of *IGFBP5* (rs1861628 and rs13020935) associated both in European Americans (p = 3.68×10^−6^ and 7.02×10^−6^, respectively) and African Americans (1.82×10^−4^ and 1.82×10^−4^, respectively). These SNPs are in perfect LD in both 1000 Genomes CEU and YRI reference panels (r^2^ = 1.00). Interestingly, while the direction of effect was consistent between the two populations, the magnitude of effect was larger in African Americans β = −0.1492, SE = 0.04; β = −0.1492, SE = 0.04, respectively) compared with European Americans (β  = −0.05, SE = 0.01; β = −0.05, SE = 0.01, respectively) (Figure 2). One additional variant, *ABO* rs657152, was significant in both European Americans (p = 4.17×10^−06^, β = 0.05) and African Americans (p = 0.03, β = 0.09). Overall, most genetic associations identified in European Americans for serum TSH levels were not significant (p<0.05) in African Americans (25/30; 83.3%); however, the majority of associations (21/30; 70.0%) had genetic effects in the same direction between the two populations (Figure 2).

### Replication and Generalization

At least 24 SNPs have been associated with serum TSH levels in European descent populations in the literature [18]–[20], [35]. We considered a SNP replicated if the direction of effect was the same as previously reported and associated at a liberal threshold of p<0.05 with serum TSH levels. In European Americans, we replicated 22/25 (88%) SNPs previously associated with serum TSH levels (Table 3). As previously mentioned, the most significant association with TSH levels in European Americans replicated the published reports for *PDE8B* SNPs rs2046045 and rs6885099 (Table 3). Beyond *PDE8B*, we replicated two SNPs on chromosome 1 in *CAPZB* previously implicated as associated with serum TSH levels (Table 3). One SNP, rs12138950, was a perfect proxy for previously-reported *CAPZB* rs10917469 (1000 Genomes CEU r^2^ = 1.00, β = −0.05, p = 8.97×10^−5^) (Table 3).

**Table 3:** Comparison of associations in eMERGE European Americans to previously published genetic associations with serum thyroid stimulating hormone (TSH) levels.

Locus			Prior Association					Current Study					
SNP	Chr	Gene/Gene Region	CA	CAF	β (SE)	P-value	Ref.	SNP/Best Proxy SNP	r^2^	CA	CAF	β (SE)	P-value
rs10917469	1	*CAPZB*	G	0.16	−0.16 (0.03)	3.2×10^−08^	[20]	rs12138950	1.00	C	0.15	−0.05 (0.01)	8.97×10^−05^
rs10917477	1	*CAPZB*	A	0.51	−0.06 (0.01)	1.54×10^−08^	[19]	rs6683419	0.73	G	0.48	0.04 (0.01)	3.56×10^−04^
rs10799824	1	*CAPZB*	A	0.16	−0.11 (0.01)	3.60×10^−21^	[15]	rs12138950	0.95	C	0.15	−0.05 (0.01)	8.97×10^−05^
rs334699	1	*NFIA*	A	0.05	−0.14 (0.02)	5.40×10^−12^	[15]	rs334708	0.79	C	0.08	−0.05 (0.02)	7.20×10^−03^
rs13015993	2	*IGFBP5*	A	0.74	0.08 (0.01)	3.24×10^−15^	[15]	rs1861628	1.00	T	0.27	−0.05 (0.01)	3.68×10^−06^
rs10028213	4	*NR3C2*	C	0.82	0.08 (0.01)	2.88×10^−10^	[19]	rs10519980	1.00	T	0.18	−0.04 (0.01)	0.001
rs10032216	4	*NR3C2*	T	0.78	0.09 (0.01)	9.28×10^−16^	[15]	rs17025017	1.00	A	0.19	−0.04 (0.01)	2.38×10^−03^
rs2046045	5	*PDE8B*	T	0.62	−0.12 (0.01)	2.79×10^−27^	[19],[36],[40]	rs2046045	--	C	0.40	0.09 (0.01)	1.85×10^−17^
rs6885099	5	*PDE8B*	A	0.59	−0.14 (0.01)	1.95×10^−56^	[15]	rs2046045	1.00	C	0.40	0.09 (0.01)	1.85×10^−17^
rs4704397	5	*PDE8B*	A	0.40*	0.21	1.64×10^−10^	[16]	rs1382879	0.94	G	0.39	0.09 (0.01)	7.16×10^−18^
rs753760	6	*PDE10A*	C	0.69	0.10 (0.01)	1.21×10^−24^	[15]	rs2983514	0.93	G	0.33	−0.05 (0.01)	1.36×10^−05^
rs9472138	6	*VEGFA*	T	0.29	−0.08 (0.01)	6.72×10^−16^	[15]	rs9472138	--	T	0.28	−0.04 (0.01)	6.41×10^−04^
rs11755845	6	*VEGFA*	T	0.27	−0.07 (0.01)	1.68×10^−10^	[15]	rs11755845	--	T	0.24	−0.02 (0.01)	0.04
rs9497965	6	*SASH1*	T	0.42	0.05 (0.01)	2.25×10^−08^	[15]	rs9377117	0.54	G	0.30	0.02 (0.01)	0.12
rs7825175	8	*NRG1*	A	0.21	−0.07 (0.01)	2.94×10^−09^	[15]	rs2466067	0.21	C	0.31	−0.05 (0.01)	8.41×10^−06^
rs657152	9	*ABO*	A	0.34	0.06 (0.01)	4.11×10^−10^	[15]	rs657152	--	T	0.38	0.05 (0.01)	4.17×10^−06^
rs1571583	9	*GLIS3*	A	0.25	0.06 (0.01)	2.55×10^−08^	[15]	rs1571583	--	T	0.25	0.03 (0.01)	0.01
rs17723470	11	*PRDM11*	T	0.28	−0.07 (0.01)	8.83×10^−11^	[15]	rs7940871	0.89	T	0.29	−0.04 (0.01)	1.42×10^−04^
rs1537424	14	*MBIP*	T	0.61	−0.05 (0.01)	1.17×10^−08^	[15]	rs1537424	--	G	0.43	0.03 (0.01)	2.89×10^−03^
rs11624776	14	*ITPK1*	A	0.66	−0.06 (0.01)	1.79×10^−09^	[15]	rs957362	0.31	C	0.22	0.02 (0.01)	0.09
rs10519227	15	*FGF7*	A	0.25	−0.07 (0.01)	1.02×10^−11^	[15]	rs7168316	1.00	T	0.23	−0.05 (0.01)	2.10×10^−05^
rs17776563	15	*MIR1179*	A	0.32	−0.06 (0.01)	2.89×10^−10^	[15]	rs11073790	0.81	T	0.35	−0.01 (0.01)	0.24
rs3813582	16	LOC440389*/MAF*	T	0.67	0.08 (0.01)	8.45×10^−18^	[19]	rs17767383	1.00	A	0.31	−0.04 (0.01)	1.42×10^−04^
rs9915657	17	*SOX9*	T	0.54	−0.06 (0.01)	7.53×10^−13^	[15]	rs9915657	--	C	0.46	0.03 (0.01)	9.53×10^−04^
rs4804416	19	*INSR*	T	0.57	−0.06 (0.01)	3.16×10^−10^	[15]	rs4804416	--	G	0.44	0.03 (0.01)	7.20×10^−04^

SNP rs number, chromosomal location, nearest gene/gene region, coded allele (CA), coded allele frequency (CAF), and association summary statistics (betas, standard errors, and p-values) are given for each previously reported association with TSH levels in European Americans. CAF for rs4704397 is the mean CAF for the combined cohorts described in Taylor et al. [16]. For SNPs not directly genotyped in this study, the proxy in highest linkage disequilibrium in 1000 Genomes CEU reference panel was identified. Results of adjusted (age, sex, body mass index, and principal component 1) tests of association are given for each previously reported SNP or its proxy in this European American dataset (n = 4,501).

In African Americans, 5/24 (25%) SNPs previously associated with TSH levels in European-descent populations generalized at a liberal significance threshold of p<0.05 and a consistent direction of effect (Table S4). *PDE8B* rs2046045, a proxy for rs6885099 (1000 Genomes CEU r^2^ = 1.00, YRI r^2^ = 0.945), was associated with serum TSH levels in African Americans (β = −0.09, p = 0.03) (Table S4). *NFIA* rs334713, a proxy for rs334699 (1000 Genomes CEU r^2^ = 1.00, YRI r^2^ = 0.774), was associated with serum TSH levels in eMERGE African Americans (p = 1.50×10^−3^) with a similar effect size (β = −0.17) as previously-reported European-descent populations. Notably, the coded allele frequency of this SNP was greater in African Americans (coded allele frequency  = 0.17; Table S4) compared with either eMERGE European Americans (0.08) or the previously-reported European descent population (0.05) (Table 3). Intronic *ABO* rs657152 was significant at p = 0.03, and the magnitude and direction of effect were similar to previously published European American data (Table S4). *VEGFA* rs11755845 was significant at p = 0.01 (Table S4) with an effect size nearly double that of the previously reported result in European Americans (Table S4). SNP rs13020935 upstream of *IGFBP5*, a proxy for rs13015993 (r^2^ = 1.00), was significant at p = 1.82×10^−4^ (Table S4).

### SNPs previously associated with thyroid disease

Next, we investigated SNPs that had previously been associated with a thyroid disease phenotype, specifically: hypothyroidism, thyroid cancer, and Graves disease [36]–[38], since variation in TSH levels may indicate thyroid disease. Six SNPs in the *FOXE1* region, including rs925489, generalized to euthyroid European American subjects (Table S5). An additional SNP in *FOXE1*, rs965513, previously associated with hypothyroidism [22],[36], generalized to serum TSH levels in European Americans (p = 1.09×10^−6^, β = −0.05) (Table S5). *FOXE1* rs1877432, previously associated with hypothyroidism, generalized to serum TSH levels in African Americans (p = 9.73×10^−3^, β = 0.11) (Table S6). *RHOH/CHRNA9* rs6832151, previously associated with Grave's Disease, generalized to serum TSH levels in African Americans (p = 0.01, β = −0.10) (Table S6). None of the SNPs previously associated with thyroid cancer [38] were associated with serum TSH levels in either European Americans or African Americans at a liberal significance threshold of p<0.05 (Tables S5 and S6). Broadly, we found little evidence of association with serum TSH levels for SNPs, apart from *FOXE1*, that have been associated with other thyroid-related phenotypes.

### Interaction with BMI

**Figure 3:**
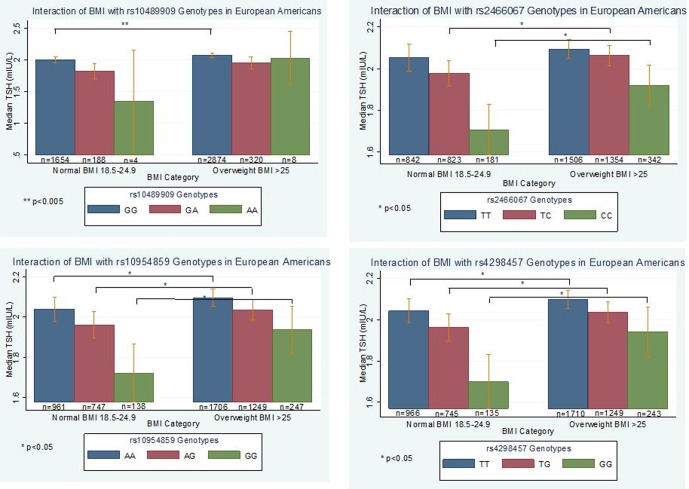
Body mass index as a modifier of serum thyroid stimulating hormone (TSH) levels genetic associations in eMERGE European Americans. Interaction analyses were performed using the SNPs with p<1×10^−04^ significance levels in the model adjusted for age, sex, PC1, and body mass index in European Americans (n = 4,501). For each significant (p<0.05) interaction term, the model was then stratified by normal/overweight BMI (normal BMI  = 18–24.9; overweight BMI ≥25). We considered a SNPxBMI interaction significant at a threshold of p<0.05. Shown are p-values from Wilcoxon rank-sum test comparing median TSH values between BMI categories at each genotype.

BMI is significantly positively associated with TSH levels and changes in BMI can be a symptom of thyroid disease, with hypothyroid persons gaining weight and hyperthyroid persons losing weight [39]. We observed that the addition of BMI into the linear regression model yielded more significant p-values for the SNPs in *PDE8B* and others, and the results from the stratified analyses differed within each race/ethnicity (Table S7, Table S8). Therefore, we performed formal tests of interaction between BMI and all SNPs (n = 118) with p<1×10^−4^ from the age, sex, PC1, and BMI adjusted model in European Americans and considered evidence for an interaction at p<0.05. Three SNPs met our significance threshold in European Americans for an interaction with BMI: *NFIA* rs10489909, *NRG1* rs2466067 and rs4298457. An additional *NRG1* SNP was just outside the p<0.05 significance threshold for the interaction: rs10954859 (Table S9, Figure 3). The *NRG1* SNPs are in moderate-to-high LD with each other (r^2^>0.70). We compared median TSH levels by BMI category for each genotype by SNP and observed lower median TSH levels for individuals with the AA genotype for rs10489909 who were of normal BMI than compared to individuals with overweight BMI (p<0.005). We observed similar trends for rs2466067 (CC genotype), rs10954859 (GG genotype), and rs4298457 (GG genotype) (p<0.05) which suggests serum TSH levels may be attenuated based on BMI for these homozygous genotypes. To understand if the observed interaction effect was a threshold effect of overweight or obese BMI, or a dose-dependent effect, we further stratified the overweight BMI category into overweight (BMI 25–30) and obese (BMI >30) in the European Americans (Figure S3). For the rs10489909, we observed lower median TSH levels for individuals with the GG genotype who were of normal BMI compared to individuals with overweight BMI (p<0.01) (Figure S3). We observed similar trends between individuals with normal BMI compared to obese BMI for rs4298457 (GG genotype) and rs2466067 (CC genotype) (p<0.05) (Figure S3). These data suggest the variation observed in serum TSH levels for these genotypes may result from a threshold-effect of obese BMI.

We also performed tests of interaction in African Americans for BMI and the 87 most significant SNPs (p<1×10^−4^ from the age, sex, PC1, and BMI adjusted model). We observed five SNPs at the p<0.05 significance threshold (Table S9, Figure S2). *MYT1L* rs6728613 and rs4073401 are in perfect LD with each other (r^2^ = 1.00) and were the most significant in this interaction analysis (p = 2.28×10^−3^) (Table S9, Figure S2). While other interaction terms were significant in the African American sample, small sample sizes and low counts made comparisons across genotypes and BMI categories difficult to interpret (Figure S2).

## Discussion

The eMERGE Network was established in 2007 to determine whether electronic medical records could be used to identify disease susceptibility in diverse patient populations for complex traits/diseases. At each study site, DNA linked to an EMR was genotyped for a GWAS for specific complex diseases (e.g., type II diabetes) and medically relevant quantitative traits (e.g., cardiac conduction). A recent eMERGE Network GWAS demonstrated that these study-specific genotype data can be “reused” for additional GWAS for binary outcomes (hypothyroidism) extracted from the EMR [22]. As an extension of this exercise, we performed a GWAS for an additional medically relevant quantitative trait: thyroid stimulating hormone (TSH) levels, in 4,501 European American and 351 African American euthyroid individuals.

Several studies have shown associations between TSH levels and *PDE8B* (briefly: [11],[35],[40],[41]). *PDE8B* is a phosphodiesterase gene that encodes a cAMP-specific protein expressed in thyroid tissue [42]. *PDE8B* upregulates cAMP through interaction with the TSH receptor on thyroid cells [11],[42]. In this study, we have replicated the results recently obtained by several groups finding association of TSH levels and several SNPs in the *PDE8B* region in European Americans [35],[40]. Variants in *PDE8B* were the only SNPs in this analysis to reach genome-wide significance in European Americans after accounting for multiple testing. In African Americans, rs2046045 (in high/perfect LD with rs6885099 and rs4704397) was nominally significant. These findings support the strong association of *PDE8B* to TSH levels in European Americans and suggest this association is generalizable to African Americans as well. Future studies to consider the association of *PDE8B* in other diverse populations are warranted.

The *FOXE1* region was not as strongly associated with TSH levels as *PDE8B* in European Americans, a result similar to that obtained by Medici et al. [40] and Alul et al. in neonates[41]. *FOXE1* encodes a thyroid transcription factor with a characteristic forkhead motif believed to be important in thyroid morphogenesis [43],[44]. Mutations in *FOXE1* have been implicated in hypothyroidism [22],[36],[38] and thyroid cancer [45],[46]. No SNPs in *FOXE1* reached genome-wide significance in this study, though several were associated at the 10^−6^ threshold in European Americans and at the 10^−3^ threshold in African Americans. As the prior association with *FOXE1* is for a disease state (hypothyroidism), it is unsurprising that we failed to find association at the genome-wide significant level in a euthyroid (non-thyroid disease) population.

Given the relationship between TSH levels and specific clinical outcomes, we hypothesized that serum TSH levels would also be associated with SNPs previously associated with hypothyroidism, Grave's Disease, or thyroid cancer by GWAS or candidate gene studies [36]–[38]. Patients with these disorders exhibit abnormal TSH levels and there is a strong autoimmune component to the diseases [30]. No SNPs in previously identified gene regions (*CTLA-4, TSHR, TTF1, HLA*, and *PTPN22*) were significantly associated with TSH levels in either European Americans or African Americans from the eMERGE Network (Tables S5 and S6), suggesting the contribution to these disorders from these genes may be specific to disease risk and not natural variation in TSH levels.

Obesity (BMI >30) has been implicated in higher TSH levels and change in an individual's set point [47],[48]. We performed additional analyses adjusting for age, sex, PC1, and BMI in both the European American and African American cohorts and stratified analyses by BMI (normal versus overweight). In the European Americans, adjusting for BMI did not appreciably modify the results, though the results in both *PDE8B* and *FOXE1* were more highly significant (Table S7). These results led us to consider potential SNPxBMI interactions. After performing tests of association for an interaction in the most significant results from the primary analysis, we identified two loci with SNPxBMI interactions in European Americans: *NFIA* and *NRG1*. *NFIA*, a transcription factor, has not previously been associated with thyroid-related traits. *NRG1* encodes neuregulin, a signaling protein recently identified in a study to be associated with thyroid cancer, potentially mediated by regulation of TSH levels [38]. Neuregulin is expressed in papillary thyroid carcinomas and has been found to regulate cell proliferation in a rat thyroid cell model [49]. Further studies on the role *NRG1* may play in regulating TSH levels are warranted. In the African American subjects, significant interactions at a liberal threshold (p<0.05) were identified, but small sample sizes and low genotype counts per BMI category made comparisons across groups difficult.

We compared results from the African Americans to those of the European Americans in our study and observed several differences. While several SNPs in *PDE8B* reached genome-wide significance in European Americans, none were significant in African Americans, and only two *PDE8B* variants identified in previous GWAS generalized to this population at a liberal significance threshold of p<0.05. Of the 32 most significant SNPs in European Americans, 21 had the same direction of effect and similar effect sizes in African Americans, suggesting the small sample size and resulting lack of power were responsible for our inability to generalize previously identified variants to the eMERGE African Americans.

A major limitation of this study is sample size. Among both populations, we excluded individuals in eMERGE with an abnormal TSH level given this study sought to identify genetic determinants of the normal distribution for TSH levels. Despite excluding individuals with abnormal TSH values, the mean (standard deviation) observed here for European Americans [1.90 (0.93)] was well within the range of previous TSH level genetic association studies: 1.5 (0.80) to 2.7 (4.1) µIU/mL [18]. The addition of the few individuals with abnormal TSH levels would unlikely increase statistical power to detect additional genome-wide associations or substantially impact the overall trait distribution. In comparison, the African American sample size was very small which impacted our ability to generalize previous findings to this population. In eMERGE African Americans, we were only adequately powered (>80%) for one test of association: *PDE8B* rs4704397. This SNP was not directly genotyped in the eMERGE African American dataset, but is in very high LD with genotyped rs2046045 in the 1000 Genomes CEU panel (r^2^ = 0.94), but not with the 1000 Genomes YRI panel (r^2^ = 0.49). The small sample size coupled with lower linkage disequilibrium resulted in underpowered tests of association for the African American dataset.

We also observed striking differences in minor allele frequencies (MAF) between European Americans and African Americans that may have impacted our ability to replicate and generalize previously associated variants (Figure 2). In European Americans, most of the minor allele frequencies were comparable to those in previously published studies (Table S10), and we were adequately powered (80%) to replicate 18/25 SNPs previously associated with serum TSH levels at a liberal significance threshold of 0.05 (Table S10). Of the 18 properly powered tests of association, all of these SNPs replicated in the eMERGE European American dataset, validating prior associations for these SNPs with TSH levels in European Americans. The utility of these variants in the clinical setting to predict serum TSH levels has not yet been calculated; future studies considering the predictive capacity of these SNPs for a clinical application may be beneficial.

This study further demonstrates the feasibility of using genotypes linked to EMRs to perform secondary analyses for quantitative traits in complex diseases in diverse populations [50],[51]. We identified SNPs associated with serum TSH levels and replicated findings from earlier GWAS for TSH levels and thyroid-related traits to the eMERGE European American euthyroid population. We further suggest BMI may modify genetic associations with serum TSH levels and that this may occur as a threshold effect with obese BMI for some genotypes. Consistent with other reports, we found few associations with SNPs associated with serum TSH levels that have effects on other thyroid-related traits/diseases, suggesting the development of thyroid disease and variation of TSH levels occurs primarily through different mechanisms. Importantly, we identified suggestive associations with biologically plausible SNPs and generalized several SNPs from previous GWAS to the eMERGE African American euthyroid population, suggesting additional studies in diverse populations are warranted.

## Supporting Information

Figure S1 **Manhattan plot of tests of association with serum TSH levels in African Americans in eMERGE.** Data shown are p-values from 905,285 single SNP tests of association for serum TSH levels in a model adjusted for age, sex, principal component (PC) 1, and body mass index in euthyroid African Americans in eMERGE Network (n = 351). Y axis represents the –log10 (p-value); horizontal lines represent Bonferroni corrected significance level (5×10−08) (top) and suggestive significance level (1×10−04) (bottom). Chromosomes are arranged on the x axis.
(TIF)

Figure S2 **Body mass index as a modifier of serum TSH levels genetic associations in eMERGE African Americans.** Interaction analyses were performed using the SNPs with p<1×10−04 significance levels in the model adjusted for age, sex, PC1, and BMI in African Americans (n = 351); the model was stratified by race/ethnicity and by normal/overweight BMI (normal: BMI 18–24.9; overweight: BMI 25+). We considered a SNPxBMI interaction significant at a threshold of p<0.05. Shown are p-values from Wilcoxon rank-sum tests comparing median TSH values between BMI categories at each genotype.
(TIF)

Figure S3 **Body mass index as a modifier of serum TSH levels genetic associations in eMERGE African Americans.** Interaction analyses were performed using the SNPs with p<1×10−4 significance levels in the model adjusted for age, sex, PC1, and BMI in European Americans (n = 4,501); the model was stratified by race/ethnicity and by normal/overweight/obese BMI (normal: BMI 18–24; overweight: BMI 25–30; obese: BMI 30+). We considered a SNPxBMI interaction significant at a threshold of p<0.05. Shown are Bonferroni-corrected p-values from multiple pairwise comparisons after ANOVA, comparing median TSH values between BMI categories at each genotype.
(TIF)

Table S1 **eMERGE Network site contributions to study participants.** Primary phenotype reflects initial GWAS phenotype investigated at each site for the eMERGE Network. Total (n) genotyped are for each site's primary phenotype GWAS. Euthyroid subjects for serum thyroid stimulating hormone (TSH) level analysis are a subset of the total number genotyped in eMERGE for the primary genotypes. All sites contributed European Americans to the serum TSH level analysis; all sites except Marshfield Clinic contributed African Americans. Data shown are counts (n).
(DOCX)

Table S2 **SNP associations for serum TSH levels in eMERGE study European Americans.** Tests of association using linear regression, adjusted for age, sex, principal component (PC1), and BMI were performed. Tests of association at p<1×10^−04^ are listed. Gene listed is the gene in closest proximity to the SNP. Coded allele frequency (CAF) is for the allele frequency in eMERGE European Americans in the serum TSH study (n = 4,501).
(DOCX)

Table S3 **SNP associations for serum TSH levels in eMERGE study African Americans.** Tests of association using linear regression, adjusted for age, sex, principal component (PC) 1, and BMI were performed. Tests of association at p<1×10^−04^ are listed. Gene listed is the gene in closest proximity to the SNP. Coded allele frequency (CAF) is for the allele frequency in eMERGE African Americans in the serum TSH study (n = 351).
(DOCX)

Table S4 **Comparison of associations in eMERGE African American TSH study participants to previously published SNP associations with serum TSH levels.** SNP rs number, chromosomal location, nearest gene/gene region, coded allele (CA), coded allele frequency (CAF), association summary statistics (betas, standard errors, and p-values), and PubMed ID (PMID) are given for each previously reported association with TSH levels in European Americans. CAF highlighted with (*) represents the average CAF in the Taylor et al. (PMID: 21317282) study. For SNPs not directly genotyped in this study, the proxy in highest linkage disequilibrium in 1000 Genomes CEU samples was identified. Results of adjusted (age, sex, BMI, and PC1) tests of association are given for each previously reported SNP or its proxy in this African American dataset (n = 351).
(DOCX)

Table S5 **Comparison of associations in eMERGE European Americans with previously published SNP associations for thyroid-related traits.** SNP rs number, chromosomal location, nearest gene/gene region, coded allele (CA), coded allele frequency (CAF), and association summary statistics (odds ratio (OR) and p-values) are given for each previously reported association with thyroid-related traits in European Americans. For SNPs not directly genotyped in this study, the proxy in highest linkage disequilibrium in 1000 Genomes CEU samples was identified. Results of adjusted (age, sex, body mass index, and principal component 1) tests of association are given for each previously reported SNP or its proxy in this European American dataset (n = ,501).
(DOCX)

Table S6 **Comparison of associations in eMERGE African Americans with previously published SNP associations for thyroid-related traits.** SNP rs number, chromosomal location, nearest gene/gene region, coded allele (CA), coded allele frequency (CAF), and association summary statistics (odds ratio (OR) and p-values) are given for each previously reported association with thyroid-related traits in European Americans. For SNPs not directly genotyped in this study, the proxy in highest linkage disequilibrium in 1000 Genomes CEU samples was identified. Results of adjusted (age, sex, body mass index, and principal component 1) tests of association are given for each previously reported SNP or its proxy in this African American dataset (n = 351).
(DOCX)

Table S7 **Comparison of SNP associations (p<10^−04^) in regression models with and without body mass index covariates for serum TSH levels in euthyroid eMERGE study European Americans (n = 4,501).** For each SNP, p-values and betas are given for models that include or exclude BMI as a covariate. All models are linear regressions assuming an additive genetic model adjusted for age, sex, and principal component 1.
(DOCX)

Table S8 **Comparison of SNP associations (p<10^−04^) in regression models with and without body mass index covariates for serum TSH levels in euthyroid eMERGE study African Americans (n = 351).** For each SNP, p-values and betas are given for models that include or exclude BMI as a covariate. All models are linear regressions assuming an additive genetic model adjusted for age, sex, and principal component 1.
(DOCX)

Table S9 **Body mass index as a modifier of serum TSH levels genetic associations.** Interaction analyses were performed using the SNPs with p<1×10^−04^ significance levels in the model adjusted for age, sex, principal component (PC) 1, and BMI in African Americans (n = 351); the model was stratified by race/ethnicity and by normal/overweight BMI (normal: BMI 18–24.9; overweight: BMI 25+). We considered a SNPxBMI interaction significant at a threshold of p<0.05. Displayed are significant interaction results at p = 0.05.
(DOCX)

Table S10 **Power calculations for replication/generalization in eMERGE TSH levels study.** Power calculations for replication/generalization of SNPs previously associated with serum TSH levels to eMERGE euthyroid European Amercians (EA) and African Americans. SNP rs number, chromosomal location, nearest gene/gene region, coded allele (CA), coded allele frequency (CAF), association summary statistics (betas and p-values), and PubMed ID (PMID) are given for each previously reported association with serum TSH levels in European Americans. Starred (*) CAF represents mean CAF from Taylor et al. Power was calculated for each race/ethnicity using Quanto assuming the previously reported effect size, an additive genetic model, a liberal significance threshold of 0.05, the eMERGE minor allele frequencies, and the eMERGE sample sizes. Power calculations labeled with an asterisk indicate proxy SNPs listed in Table 3 (European Americans) and Table S4 (African Americans) as described in the Methods.
(DOCX)
