# Supplementary figures and images for: Genetic Variants Associated with Serum Thyroid Stimulating Hormone (TSH) Levels in European Americans and African Americans from the eMERGE Network
Source: PLoS One. 2014 Dec 1;9(12):e111301. doi: 10.1371/journal.pone.0111301 (PMC4249871; doi:10.1371/journal.pone.0111301)

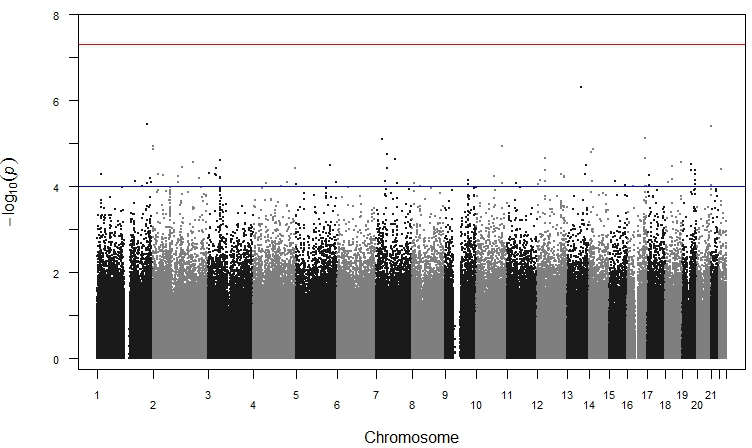

Supplement: Figure S1 — Manhattan plot of tests of association with serum TSH levels in African Americans in eMERGE. Data shown are p-values from 905,285 single SNP tests of association for serum TSH levels in a model adjusted for age, sex, principal component (PC) 1, and body mass index in euthyroid African Americans in eMERGE Network (n = 351). Y axis represents the –log10 (p-value); horizontal lines represent Bonferroni corrected significance level (5×10^−08^) (top) and suggestive significance level (1×10^−04^) (bottom). Chromosomes are arranged on the x axis. (TIF) [file pone.0111301.s001.tif]

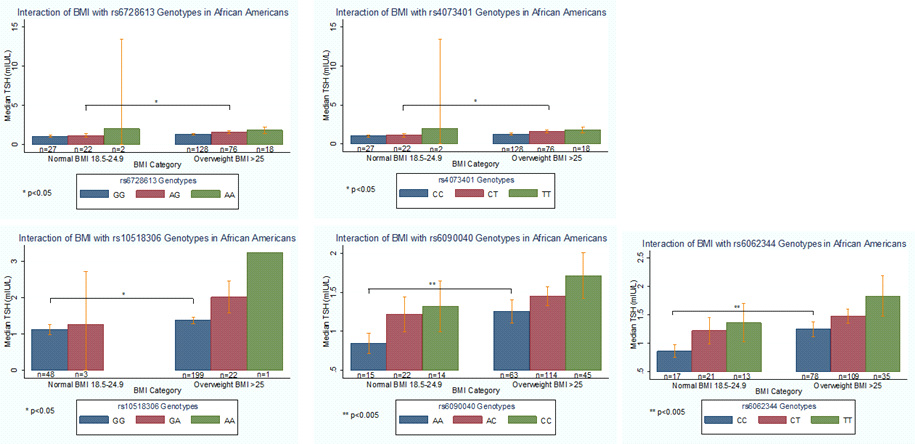

Supplement: Figure S2 — Body mass index as a modifier of serum TSH levels genetic associations in eMERGE African Americans. Interaction analyses were performed using the SNPs with p<1×10^−04^ significance levels in the model adjusted for age, sex, PC1, and BMI in African Americans (n = 351); the model was stratified by race/ethnicity and by normal/overweight BMI (normal: BMI 18–24.9; overweight: BMI 25+). We considered a SNPxBMI interaction significant at a threshold of p<0.05. Shown are p-values from Wilcoxon rank-sum tests comparing median TSH values between BMI categories at each genotype. (TIF) [file pone.0111301.s002.tif]

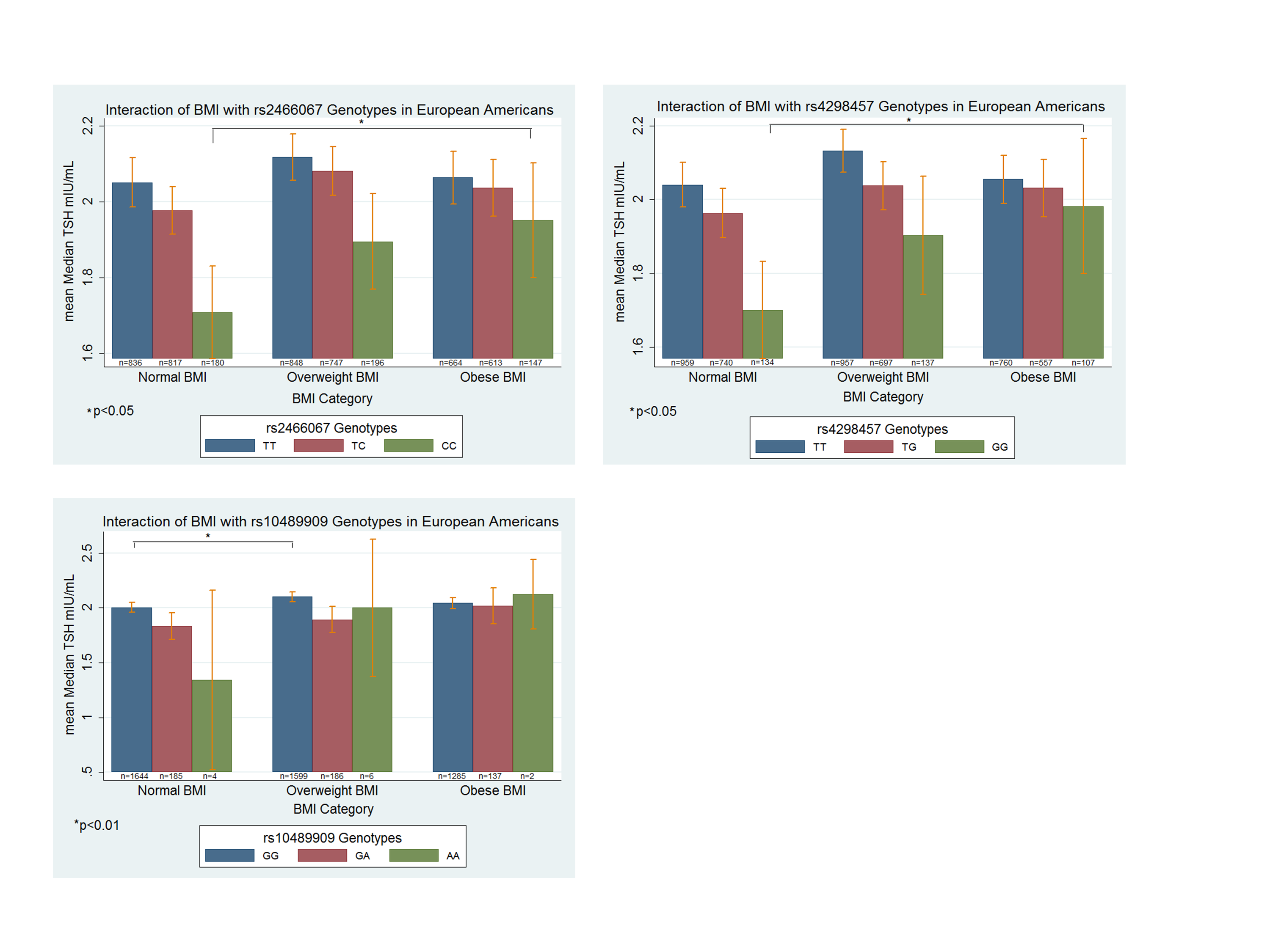

Supplement: Figure S3 — Body mass index as a modifier of serum TSH levels genetic associations in eMERGE African Americans. Interaction analyses were performed using the SNPs with p<1×10^−4^ significance levels in the model adjusted for age, sex, PC1, and BMI in European Americans (n = 4,501); the model was stratified by race/ethnicity and by normal/overweight/obese BMI (normal: BMI 18–24; overweight: BMI 25–30; obese: BMI 30+). We considered a SNPxBMI interaction significant at a threshold of p<0.05. Shown are Bonferroni-corrected p-values from multiple pairwise comparisons after ANOVA, comparing median TSH values between BMI categories at each genotype. (TIF) [file pone.0111301.s003.tif]
