# Supplementary material for: Genetic Variants Associated with Serum Thyroid Stimulating Hormone (TSH) Levels in European Americans and African Americans from the eMERGE Network
Source: PLoS One. 2014 Dec 1;9(12):e111301. doi: 10.1371/journal.pone.0111301 (PMC4249871; doi:10.1371/journal.pone.0111301)
Supplement: Table S1 — eMERGE Network site contributions to study participants. Primary phenotype reflects initial GWAS phenotype investigated at each site for the eMERGE Network. Total (n) genotyped are for each site's primary phenotype GWAS. Euthyroid subjects for serum thyroid stimulating hormone (TSH) level analysis are a subset of the total number genotyped in eMERGE for the primary genotypes. All sites contributed European Americans to the serum TSH level analysis; all sites except Marshfield Clinic contributed African Americans. Data shown are counts (n). (DOCX) [file pone.0111301.s004.docx]

**Table S1: eMERGE Network site contributions to study participants.** Primary phenotype reflects initial GWAS phenotype investigated at each site for the eMERGE Network. Total (n) genotyped are for each site’s primary phenotype GWAS. Euthyroid subjects for serum thyroid stimulating hormone (TSH) level analysis are a subset of the total number genotyped in eMERGE for the primary genotypes. All sites contributed European Americans to the serum TSH level analysis; all sites except Marshfield Clinic contributed African Americans. Data shown are counts (n).

| **Site** | **Primary Phenotype** | **Total # Genotyped (n)** | **TSH Levels** | |
| --- | --- | --- | --- | --- |
|  |  |  | **European Americans**  **(n)** | **African Americans**  **(n)** |
| Marshfield Clinic | Cataracts | 4,113 | 1,157 | 0 |
| Vanderbilt | Cardiac Conductance | 2,712 | 284 | 88 |
| Group Health | Dementia | 2,532 | 1,167 | 64 |
| Mayo Clinic | Peripheral Artery Disease | 3,043 | 1,881 | 10 |
| Northwestern | Type 2 Diabetes | 1,217 | 12 | 189 |
| **Total** |  | **13,617** | **4,501** | **351** |
