# Supplementary material for: Genetic Variants Associated with Serum Thyroid Stimulating Hormone (TSH) Levels in European Americans and African Americans from the eMERGE Network
Source: PLoS One. 2014 Dec 1;9(12):e111301. doi: 10.1371/journal.pone.0111301 (PMC4249871; doi:10.1371/journal.pone.0111301)
Supplement: Table S2 — SNP associations for serum TSH levels in eMERGE study European Americans. Tests of association using linear regression, adjusted for age, sex, principal component (PC1), and BMI were performed. Tests of association at p<1×10^−04^ are listed. Gene listed is the gene in closest proximity to the SNP. Coded allele frequency (CAF) is for the allele frequency in eMERGE European Americans in the serum TSH study (n = 4,501). (DOCX) [file pone.0111301.s005.docx]

**Table S2: SNP associations for serum TSH levels in eMERGE study European Americans.** Tests of association using linear regression, adjusted for age, sex, principal component (PC1), and BMI were performed. Tests of association at p<1x10^-04^ are listed. Gene listed is the gene in closest proximity to the SNP. Coded allele frequency (CAF) is for the allele frequency in eMERGE European Americans in the serum TSH study (n=4,501).

| **CHR** | **SNP** | **GENE** | **GENE REGION** | **CODED ALLELE** | **CAF** | **BETA (SE)** | **P-VALUE** |
| --- | --- | --- | --- | --- | --- | --- | --- |
| 5 | rs1382879 | *PDE8B* | intronic | G | 0.39 | 0.09 (0.01) | 7.16E-18 |
| 5 | rs2046045 | *PDE8B* | intronic | C | 0.40 | 0.09 (0.01) | 1.85E-17 |
| 5 | rs989758 | *PDE8B* | intronic | T | 0.36 | 0.08 (0.01) | 1.33E-14 |
| 5 | rs9687206 | *PDE8B* | intronic | G | 0.43 | 0.08 (0.01) | 5.52E-14 |
| 5 | rs12515498 | *PDE8B* | intronic | C | 0.26 | 0.07 (0.01) | 3.27E-10 |
| 5 | rs6885813 | *PDE8B* | intronic | A | 0.25 | 0.06 (0.01) | 4.05E-08 |
| 5 | rs1096752 | *PDE8B* | intronic | A | 0.45 | -0.05 (0.01) | 6.30E-07 |
| 5 | rs13361710 | *PDE8B* | intronic | T | 0.24 | 0.06 (0.01) | 6.60E-07 |
| 9 | rs10759944 | *FOXE1* | upstream | A | 0.33 | -0.05 (0.01) | 1.08E-06 |
| 9 | rs965513 | *FOXE1* | upstream | A | 0.34 | -0.05 (0.01) | 1.09E-06 |
| 9 | rs925489 | *FOXE1* | upstream | C | 0.34 | -0.05 (0.01) | 1.79E-06 |
| 9 | rs7850258 | *FOXE1* | upstream | A | 0.33 | -0.05 (0.01) | 1.85E-06 |
| 2 | rs10496992 | - | intergenic | G | 0.38 | 0.05 (0.01) | 2.22E-06 |
| 2 | rs1861628 | *IGFBP5* | upstream | T | 0.27 | -0.05 (0.01) | 3.68E-06 |
| 5 | rs4348174 | *ITGA1* | upstream | C | 0.40 | 0.05 (0.01) | 3.97E-06 |
| 9 | rs657152 | *ABO* | intronic | T | 0.38 | 0.05 (0.01) | 4.18E-06 |
| 7 | rs740083 | *VWC2* | upstream | A | 0.24 | -0.05 (0.01) | 4.56E-06 |
| 7 | rs813379 | *CDK14* | intronic | G | 0.06 | -0.10 (0.02) | 4.57E-06 |
| 2 | rs2712168 | *IGFBP5* | upstream | C | 0.13 | 0.07 (0.01) | 4.98E-06 |
| 5 | rs256438 | *THBS4* | intronic | C | 0.36 | 0.05 (0.01) | 5.53E-06 |
| 18 | rs4570936 | - | intergenic | T | 0.22 | -0.05 (0.01) | 5.73E-06 |
| 2 | rs6546537 | *AAK1* | intronic | C | 0.28 | -0.05 (0.01) | 5.92E-06 |
| 9 | rs7855088 | *ANP32B* | upstream | C | 0.44 | -0.05 (0.01) | 6.23E-06 |
| 9 | rs925487 | *FOXE1* | downstream | G | 0.37 | -0.05 (0.01) | 6.24E-06 |
| 7 | rs803174 | *CDK14* | intronic | G | 0.06 | -0.10 (0.02) | 6.74E-06 |
| 5 | rs2438632 | *THBS4* | downstream | A | 0.39 | 0.05 (0.01) | 6.88E-06 |
| 2 | rs13020935 | *IGFBP5* | upstream | G | 0.28 | -0.05 (0.01) | 7.02E-06 |
| 5 | rs12520862 | *PDE8B* | intronic | T | 0.14 | 0.06 (0.01) | 7.48E-06 |
| 9 | rs10984103 | *FOXE1* | downstream | A | 0.37 | -0.05 (0.01) | 7.81E-06 |
| 9 | rs907580 | *FOXE1* | downstream | A | 0.27 | -0.05 (0.01) | 8.20E-06 |
| 8 | rs2466067 | *NRG1* | intronic | C | 0.31 | -0.05 (0.01) | 8.42E-06 |
| 9 | rs7870926 | *ANP32B* | downstream | G | 0.50 | -0.04 (0.01) | 8.67E-06 |
| 5 | rs7341064 | *ITGA1* | upstream | C | 0.40 | 0.04 (0.01) | 1.03E-05 |
| 8 | rs4298457 | *NRG1* | intronic | G | 0.27 | -0.05 (0.01) | 1.07E-05 |
| 11 | rs598599 | *MRE11A* | intronic | A | 0.28 | 0.05 (0.01) | 1.09E-05 |
| 4 | rs4693596 | *COQ2* | intronic | C | 0.38 | -0.04 (0.01) | 1.10E-05 |
| 8 | rs10954859 | *NRG1* | intronic | G | 0.27 | -0.05 (0.01) | 1.12E-05 |
| 5 | rs404375 | *THBS4* | intronic | G | 0.50 | -0.04 (0.01) | 1.26E-05 |
| 6 | rs2983525 | *PDE10A* | intronic | C | 0.27 | -0.05 (0.01) | 1.34E-05 |
| 6 | rs2983514 | *PDE10A* | intronic | G | 0.33 | -0.05 (0.01) | 1.36E-05 |
| 1 | rs3766122 | *SELP* | intronic | C | 0.05 | -0.10 (0.02) | 1.42E-05 |
| 9 | rs7866436 | *C9orf156* | downstream | G | 0.37 | -0.04 (0.01) | 1.52E-05 |
| 9 | rs7024345 | *FOXE1* | upstream | A | 0.27 | -0.05 (0.01) | 1.65E-05 |
| 5 | rs26367 | *FSTL4* | intronic | G | 0.10 | -0.07 (0.02) | 1.65E-05 |
| 5 | rs10073636 | *HCN1* | intronic | T | 0.43 | 0.04 (0.01) | 1.73E-05 |
| 9 | rs13285674 | *ASTN2* | upstream | A | 0.23 | 0.05 (0.01) | 1.90E-05 |
| 9 | rs505922 | *ABO* | intronic | C | 0.36 | 0.04 (0.01) | 1.94E-05 |
| 5 | rs7445986 | *ITGA1* | upstream | T | 0.40 | 0.04 (0.01) | 1.95E-05 |
| 2 | rs10204522 | *IGFBP5* | upstream | C | 0.10 | 0.07 (0.02) | 1.95E-05 |
| 6 | rs4054489 | *IBTK* | downstream | T | 0.18 | -0.05 (0.01) | 1.95E-05 |
| 11 | rs1055075 | *TTC12* | downstream | T | 0.34 | -0.04 (0.01) | 1.96E-05 |
| 4 | rs4861534 | *DCTD* | downstream | G | 0.10 | 0.07 (0.02) | 2.09E-05 |
| 15 | rs7168316 | *C15orf33* | intronic | T | 0.23 | -0.05 (0.01) | 2.10E-05 |
| 9 | rs7848973 | *FOXE1* | upstream | A | 0.40 | -0.04 (0.01) | 2.11E-05 |
| 12 | rs3136559 | *CD69* | upstream | A | 0.28 | 0.05 (0.01) | 2.13E-05 |
| 2 | rs6727435 | *AAK1* | intronic | A | 0.27 | -0.05 (0.01) | 2.15E-05 |
| 5 | rs33613 | *FSTL4* | intronic | T | 0.09 | -0.07 (0.02) | 2.35E-05 |
| 15 | rs12592277 | *C15orf33* | intronic | A | 0.22 | -0.05 (0.01) | 2.35E-05 |
| 8 | rs2466062 | *NRG1* | intronic | G | 0.30 | -0.05 (0.01) | 2.36E-05 |
| 8 | rs3898456 | *FAM135B* | intronic | A | 0.35 | 0.04 (0.01) | 2.56E-05 |
| 3 | rs4402960 | *IGF2BP2* | intronic | T | 0.30 | -0.05 (0.01) | 2.63E-05 |
| 3 | rs1470579 | *IGF2BP2* | intronic | C | 0.31 | -0.05 (0.01) | 2.67E-05 |
| 5 | rs13354798 | *HCN1* | intronic | C | 0.43 | 0.04 (0.01) | 2.75E-05 |
| 5 | rs9686502 | *PDE8B* | intronic | G | 0.49 | 0.04 (0.01) | 2.85E-05 |
| 22 | rs9606756 | *PDE8B* | intronic | G | 0.12 | 0.07 (0.02) | 2.86E-05 |
| 11 | rs494442 | *KIRREL3* | upstream | T | 0.40 | -0.04 (0.01) | 3.03E-05 |
| 12 | rs2695148 | *ANAPC5* | upstream | T | 0.10 | -0.07 (0.02) | 3.11E-05 |
| 1 | rs17265852 | *NFIA* | intronic | C | 0.08 | -0.07 (0.02) | 3.16E-05 |
| 5 | rs6414906 | *HCN1* | intronic | C | 0.43 | 0.04 (0.01) | 3.51E-05 |
| 16 | rs3813583 | *WWOX* | downstream | C | 0.38 | 0.04 (0.01) | 4.06E-05 |
| 1 | rs749378 | *GLIS1* | downstream | A | 0.27 | -0.05 (0.01) | 4.09E-05 |
| 5 | rs6451801 | *HCN1* | intronic | A | 0.43 | 0.04 (0.01) | 4.10E-05 |
| 5 | rs13162651 | *HCN1* | intronic | C | 0.43 | 0.04 (0.01) | 4.11E-05 |
| 6 | rs12201217 | *CDKAL1* | intronic | T | 0.38 | -0.04 (0.01) | 4.30E-05 |
| 3 | rs370234 | *VGLL4* | upstream | T | 0.39 | -0.04 (0.01) | 4.32E-05 |
| 12 | rs1647253 | *ANAPC5* | upstream | A | 0.10 | -0.07 (0.02) | 4.57E-05 |
| 8 | rs6989877 | *NRG1* | downstream | T | 0.13 | 0.06 (0.01) | 4.59E-05 |
| 6 | rs11963665 | *FAM46A* | upstream | C | 0.20 | -0.05 (0.01) | 4.63E-05 |
| 5 | rs6892290 | *HCN1* | intronic | G | 0.43 | 0.04 (0.01) | 4.76E-05 |
| 1 | rs6668505 | *PTAFR* | intronic | T | 0.06 | -0.08 (0.02) | 4.88E-05 |
| 19 | rs3745746 | *CABP5* | missense | C | 0.39 | -0.04 (0.01) | 4.93E-05 |
| 5 | rs12521494 | *PDE8B* | intronic | C | 0.23 | 0.05 (0.01) | 5.00E-05 |
| 5 | rs10064949 | *ITGA1* | upstream | C | 0.43 | 0.04 (0.01) | 5.07E-05 |
| 2 | rs1515259 | - | intergenic | T | 0.45 | 0.04 (0.01) | 5.44E-05 |
| 2 | rs1012319 | *IGFBP5* | upstream | T | 0.19 | -0.05 (0.01) | 5.47E-05 |
| 6 | rs2983500 | *PDE10A* | intronic | T | 0.11 | -0.06 (0.02) | 5.54E-05 |
| 18 | rs8096947 | - | intergenic | A | 0.19 | -0.05 (0.01) | 5.56E-05 |
| 2 | rs888186 | *IGFBP5* | upstream | C | 0.10 | -0.07 (0.02) | 5.63E-05 |
| 5 | rs4703797 | *THBS4* | intronic | G | 0.33 | 0.04 (0.01) | 5.65E-05 |
| 11 | rs529126 | *MRE11A* | intronic | A | 0.26 | 0.04 (0.01) | 5.65E-05 |
| 1 | rs11805172 | *SESN2* | upstream | G | 0.07 | -0.08 (0.02) | 5.69E-05 |
| 14 | rs8009673 | *ARHGAP5* | upstream | A | 0.15 | 0.06 (0.01) | 5.83E-05 |
| 11 | rs1939422 | *C11orf87* | upstream | T | 0.36 | -0.04 (0.01) | 5.94E-05 |
| 2 | rs1986415 | *AOX1* | intronic | A | 0.12 | 0.06 (0.02) | 6.05E-05 |
| 8 | rs2439300 | *NRG1* | intronic | A | 0.27 | -0.04 (0.01) | 6.19E-05 |
| 8 | rs2943179 | *CNBD1* | intronic | T | 0.22 | 0.05 (0.01) | 6.31E-05 |
| 15 | rs8035662 | *MEGF11* | intronic | A | 0.33 | -0.04 (0.10) | 6.37E-05 |
| 11 | rs877138 | *ANKK1* | upstream | G | 0.35 | -0.04 (0.01) | 6.37E-05 |
| 9 | rs1443434 | *FOXE1* | UTR-3’ | G | 0.40 | -0.04 (0.01) | 6.53E-05 |
| 2 | rs2381866 | - | intergenic | C | 0.44 | 0.04 (0.01) | 6.68E-05 |
| 2 | rs888182 | *IGFBP5* | upstream | C | 0.16 | 0.05 (0.01) | 6.75E-05 |
| 16 | rs7184757 | *WWOX* | intronic | C | 0.09 | -0.07 (0.02) | 7.22E-05 |
| 12 | rs11172482 | *XRCC6BP1* | downstream | C | 0.37 | -0.04 (0.01) | 7.29E-05 |
| 7 | rs39334 | *RELN* | intronic | G | 0.37 | 0.04 (0.01) | 7.47E-05 |
| 11 | rs12278001 | *DDX10* | downstream | A | 0.06 | -0.08 (0.02) | 7.53E-05 |
| 5 | rs12654213 | *HCN1* | upstream | G | 0.43 | 0.04 (0.01) | 7.69E-05 |
| 1 | rs10489909 | *NFIA* | intronic | A | 0.05 | -0.09 (0.02) | 7.81E-05 |
| 7 | rs13231383 | *TPK1* | upstream | A | 0.25 | 0.04 (0.01) | 8.19E-05 |
| 5 | rs2306344 | *PDE8B* | intronic | A | 0.31 | -0.04 (0.01) | 8.23E-05 |
| 19 | rs11666426 | *ZNF665* | intronic | C | 0.41 | 0.04 (0.01) | 8.30E-05 |
| 1 | rs12138950 | *CAPZB* | upstream | C | 0.15 | -0.05 (0.01) | 8.97E-05 |
| 9 | rs424829 | *STOM* | upstream | A | 0.29 | 0.04 (0.01) | 9.02E-05 |
| 15 | rs11071858 | *MEGF11* | intronic | G | 0.41 | -0.04 (0.01) | 9.33E-05 |
| 11 | rs12282135 | *OR52E2* | upstream | C | 0.15 | -0.05 (0.01) | 9.47E-05 |
| 1 | rs11118832 | *DUSP10* | intronic | C | 0.08 | -0.07 (0.02) | 9.52E-05 |
| 1 | rs630505 | *DENND2D* | intronic | C | 0.27 | -0.04 (0.01) | 9.58E-05 |
| 2 | rs16856529 | *IGFBP5* | upstream | C | 0.15 | 0.05 (0.01) | 9.80E-05 |
| 12 | rs1502816 | *XRCC6BP1* | downstream | C | 0.38 | -0.04 (0.01) | 9.95E-05 |
