# Supplementary material for: Genetic Variants Associated with Serum Thyroid Stimulating Hormone (TSH) Levels in European Americans and African Americans from the eMERGE Network
Source: PLoS One. 2014 Dec 1;9(12):e111301. doi: 10.1371/journal.pone.0111301 (PMC4249871; doi:10.1371/journal.pone.0111301)
Supplement: Table S3 — SNP associations for serum TSH levels in eMERGE study African Americans. Tests of association using linear regression, adjusted for age, sex, principal component (PC) 1, and BMI were performed. Tests of association at p<1×10^−04^ are listed. Gene listed is the gene in closest proximity to the SNP. Coded allele frequency (CAF) is for the allele frequency in eMERGE African Americans in the serum TSH study (n = 351). (DOCX) [file pone.0111301.s006.docx]

**Table S3: SNP associations for serum TSH levels in eMERGE study African Americans.** Tests of association using linear regression, adjusted for age, sex, principal component (PC) 1, and BMI were performed. Tests of association at p<1x10^-04^ are listed. Gene listed is the gene in closest proximity to the SNP. Coded allele frequency (CAF) is for the allele frequency in eMERGE African Americans in the serum TSH study (n=351).

| **CHR** | **SNP** | **GENE** | **GENE REGION** | **CODED ALLELE** | **CAF** | **BETA (SE)** | **P VALUE** |
| --- | --- | --- | --- | --- | --- | --- | --- |
| 13 | rs1409005 | *POU4F1-AS1* | downstream-POU4F1 | T | 0.20 | 0.25 (0.05) | 5.02E-07 |
| 1 | rs2378497 | *DUSP10* | upstream | G | 0.08 | 0.33 (0.07) | 3.53E-06 |
| 20 | rs6062344 | *TCEA2* | intronic | T | 0.40 | 0.18 (0.04) | 4.06E-06 |
| 16 | rs270421 | *WWOX* | downstream | C | 0.28 | 0.19 (0.04) | 7.75E-06 |
| 7 | rs2299116 | *CREB5* | intronic | A | 0.17 | 0.25 (0.06) | 8.16E-06 |
| 2 | rs6728613 | *MYT1L* | intronic | A | 0.24 | 0.20 (0.04) | 1.14E-05 |
| 10 | rs6585018 | *PDCD4* | near-5' | G | 0.17 | -0.22 (0.05) | 1.17E-05 |
| 14 | rs1013757 | - | downstream-TTC6, | A | 0.32 | -0.19 (0.04) | 1.33E-05 |
| 2 | rs4073401 | *MYT1L* | intronic | T | 0.24 | 0.19 (0.04) | 1.33E-05 |
| 14 | rs12883861 | - | downstream-LOC728755 | G | 0.20 | 0.21 (0.05) | 1.63E-05 |
| 7 | rs9784959 | *ABCA13* | intronic | A | 0.30 | -0.18 (0.04) | 1.82E-05 |
| 16 | rs270422 | *WWOX* | downstream | A | 0.29 | 0.18 (0.04) | 2.17E-05 |
| 12 | rs261875 | *BICD1* | intronic | C | 0.32 | 0.18 (0.04) | 2.24E-05 |
| 7 | rs274614 | *GRM3* | intronic | G | 0.30 | -0.18 (0.04) | 2.36E-05 |
| 3 | rs11711934 | *DNAH1* | intronic | C | 0.31 | -0.17 (0.04) | 2.45E-05 |
| 2 | rs12621889 | *KIAA1715* | intronic | T | 0.06 | 0.36 (0.08) | 2.68E-05 |
| 2 | rs12464144 | *KIAA1715* | intronic | A | 0.06 | 0.36 (0.08) | 2.68E-05 |
| 18 | rs10163845 | *NETO1* | near-5' | A | 0.28 | -0.18 (0.04) | 2.74E-05 |
| 19 | rs12610504 | *ZNF536* | downstream | G | 0.19 | 0.20 (0.05) | 3.07E-05 |
| 13 | rs1274744 | - | intergenic | C | 0.42 | -0.17 (0.04) | 3.21E-05 |
| 5 | rs10060607 | *SLC36A3* | intronic | A | 0.30 | 0.18 (0.04) | 3.28E-05 |
| 18 | rs1824304 | *FAM59A* | intronic | C | 0.37 | 0.17 (0.04) | 3.32E-05 |
| 2 | rs841452 | *HS6ST1* | upstream | C | 0.37 | 0.17 (0.04) | 3.52E-05 |
| 7 | rs11977108 | *ABCA13* | intronic | A | 0.17 | -0.21 (0.05) | 3.70E-05 |
| 3 | rs4678798 | *ARPP21* | intronic | A | 0.14 | 0.24 (0.06) | 3.71E-05 |
| 4 | rs6851816 | *MLF1IP* | intronic | T | 0.50 | 0.16 (0.04) | 3.83E-05 |
| 22 | rs133201 | *LRP5L* | 5'-untranslated region | A | 0.09 | 0.27 (0.06) | 4.04E-05 |
| 12 | rs2593996 | *BICD1* | intronic | C | 0.50 | -0.16 (0.04) | 4.09E-05 |
| 19 | rs1054713 | *KLK1* | cds-synon | T | 0.26 | 0.19 (0.05) | 4.16E-05 |
| 19 | rs12609319 | *ZNF536* | downstream | T | 0.19 | 0.20 (0.05) | 4.23E-05 |
| 3 | rs1918092 | - | downstream-*ARL8B*, downstream-*EDEM1* | C | 0.09 | 0.30 (0.07) | 4.90E-05 |
| 12 | rs2303478 | *ASCL4* | downstream | A | 0.28 | 0.18 (0.04) | 5.11E-05 |
| 1 | rs3738605 | *SZRD1* | 3'-untranslated region | A | 0.12 | 0.24 (0.06) | 5.12E-05 |
| 19 | rs2659099 | *MGC45922* | near-5' | T | 0.29 | 0.18 (0.04) | 5.13E-05 |
| 3 | rs4955261 | *CMTM8* | upstream | G | 0.39 | 0.16 (0.04) | 5.19E-05 |
| 13 | rs4772145 | *DOCK9* | downstream | T | 0.43 | 0.15 (0.04) | 5.23E-05 |
| 2 | rs13403407 | *C2orf43* | intronic | C | 0.47 | -0.16 (0.04) | 5.31E-05 |
| 3 | rs1513476 | *ARPP21* | intronic | C | 0.14 | 0.22 (0.05) | 5.46E-05 |
| 2 | rs17032566 | *CAMKMT* | intronic | T | 0.07 | -0.30 (0.07) | 5.52E-05 |
| 17 | rs1105813 | *DNAH2* | intronic | T | 0.43 | 0.16 (0.04) | 5.61E-05 |
| 12 | rs1563333 | *DYNLL1* | intronic | A | 0.22 | -0.19 (0.05) | 5.64E-05 |
| 10 | rs1907356 | *C10orf11* | intronic | T | 0.16 | -0.21 (0.05) | 5.71E-05 |
| 10 | rs11001788 | *C10orf11* | intronic | A | 0.16 | -0.21 (0.05) | 5.71E-05 |
| 2 | rs12470895 | *LOC729164* | ncRNA | T | 0.20 | 0.21 (0.05) | 5.86E-05 |
| 3 | rs646929 | *CACNA2D3* | intronic | C | 0.08 | 0.30 (0.07) | 5.96E-05 |
| 3 | rs2335640 | *DNAH1* | intronic | C | 0.30 | -0.17 (0.04) | 5.98E-05 |
| 12 | rs3742049 | *COQ5* | missense | T | 0.25 | 0.18 (0.04) | 6.08E-05 |
| 3 | rs17052068 | *DNAH1* | intronic | T | 0.30 | -0.16 (0.04) | 6.46E-05 |
| 1 | rs2819757 | *RYR2* | intronic | C | 0.18 | 0.22 (0.05) | 6.49E-05 |
| 2 | rs10804139 | *PARD3B* | upstream | A | 0.38 | -0.16 (0.04) | 6.54E-05 |
| 18 | rs736218 | *FAM59A* | intronic | C | 0.38 | 0.16 (0.04) | 6.66E-05 |
| 9 | rs10989120 | *MSANTD3-TMEFF1* | intronic | A | 0.39 | -0.19 (0.05) | 7.05E-05 |
| 12 | rs10744020 | *C12orf36* | downstream | C | 0.23 | 0.16 (0.04) | 7.05E-05 |
| 19 | rs2659103 | *KLK1* | intronic | T | 0.25 | 0.19 (0.05) | 7.29E-05 |
| 1 | rs10918914 | *XCL2* | downstream | G | 0.17 | 0.22 (0.05) | 7.39E-05 |
| 12 | rs261878 | *BICD1* | intronic | C | 0.32 | -0.16 (0.04) | 7.42E-05 |
| 15 | rs12914266 | *SQRDL* | intronic | A | 0.29 | 0.17 (0.04) | 7.58E-05 |
| 7 | rs6965055 | *C7orf10* | intronic | G | 0.39 | -0.16 (0.04) | 7.65E-05 |
| 7 | rs7808606 | *C7orf10* | intronic | C | 0.39 | -0.15 (0.04) | 7.66E-05 |
| 14 | rs17322359 | *PRKD1* | upstream | T | 0.10 | 0.25 (0.06) | 7.74E-05 |
| 5 | rs11949641 | *MSX2* | downstream | A | 0.23 | 0.18 (0.05) | 7.89E-05 |
| 1 | rs12120382 | *CHRM3* | upstream | C | 0.09 | 0.29 (0.07) | 7.96E-05 |
| 2 | rs6731363 | *LOC729164* | ncRNA | A | 0.20 | 0.20 (0.05) | 7.99E-05 |
| 4 | rs13144021 | *NR3C2* | upstream | G | 0.14 | 0.23 (0.06) | 8.00E-05 |
| 18 | rs877128 | *MC2R* | intronic | A | 0.25 | 0.18 (0.04) | 8.10E-05 |
| 10 | rs7923004 | *BBIP1* | intronic | C | 0.18 | -0.20 (0.05) | 8.19E-05 |
| 8 | rs6999969 | *XKR6* | intronic | C | 0.42 | -0.16 (0.04) | 8.33E-05 |
| 11 | rs1027388 | *LRRC4C* | intronic | A | 0.28 | -0.17 (0.04) | 8.36E-05 |
| 1 | rs17011253 | - | upstream-DUSP10 | C | 0.09 | 0.27 (0.07) | 8.38E-05 |
| 10 | rs942077 | *RBM20* | missense | G | 0.48 | -0.15 (0.04) | 8.47E-05 |
| 4 | rs4370216 | INTERGENIC |  | C | 0.46 | -0.15 (0.04) | 8.55E-05 |
| 4 | rs2333727 | *HSFY2* | upstream | C | 0.46 | -0.15 (0.04) | 8.55E-05 |
| 7 | rs1029357 | *SAMD9L* | 3'-untranslated region | G | 0.46 | 0.15 (0.04) | 8.57E-05 |
| 9 | rs1332598 | *MSANTD3-TMEFF1* | intronic | A | 0.23 | -0.19 (0.05) | 8.69E-05 |
| 5 | rs6864667 | *SLC12A7* | intronic | G | 0.47 | 0.15 (0.04) | 8.98E-05 |
| 12 | rs4411338 | *CCND2* | upstream | C | 0.29 | 0.16 (0.04) | 9.05E-05 |
| 19 | rs171953 | *KLK1* | downstream | G | 0.48 | -0.15 (0.04) | 9.07E-05 |
| 19 | GA035020 | *SSC5D* | intronic | T | 0.25 | 0.19 (0.05) | 9.11E-05 |
| 15 | rs2040578 | *SV2B* | intronic | G | 0.29 | 0.17 (0.04) | 9.13E-05 |
| 17 | rs1106826 | *DNAH2* | intronic | A | 0.32 | 0.17 (0.04) | 9.23E-05 |
| 20 | rs6090040 | *TCEA2* | intronic | C | 0.47 | 0.15 (0.04) | 9.35E-05 |
| 20 | rs4408777 | *RGS19* | intronic | G | 0.33 | 0.16 (0.04) | 9.39E-05 |
| 16 | rs2521676 | - | intergenic | G | 0.39 | 0.16 (0.04) | 9.73E-05 |
| 1 | rs16845412 | - | intergenic | G | 0.10 | 0.27 (0.07) | 9.76E-05 |
| 4 | rs10518306 | *LOC285419* | intronic | A | 0.06 | 0.35 (0.09) | 9.78E-05 |
| 8 | rs10098991 | - | intergenic | C | 0.44 | 0.16 (0.04) | 9.86E-05 |
| 16 | rs8059691 | *EMC8* | intronic | G | 0.11 | 0.23 (0.06) | 9.90E-05 |
