# Supplementary material for: Genetic Variants Associated with Serum Thyroid Stimulating Hormone (TSH) Levels in European Americans and African Americans from the eMERGE Network
Source: PLoS One. 2014 Dec 1;9(12):e111301. doi: 10.1371/journal.pone.0111301 (PMC4249871; doi:10.1371/journal.pone.0111301)
Supplement: Table S4 — Comparison of associations in eMERGE African American TSH study participants to previously published SNP associations with serum TSH levels. SNP rs number, chromosomal location, nearest gene/gene region, coded allele (CA), coded allele frequency (CAF), association summary statistics (betas, standard errors, and p-values), and PubMed ID (PMID) are given for each previously reported association with TSH levels in European Americans. CAF highlighted with (*) represents the average CAF in the Taylor et al. (PMID: 21317282) study. For SNPs not directly genotyped in this study, the proxy in highest linkage disequilibrium in 1000 Genomes CEU samples was identified. Results of adjusted (age, sex, BMI, and PC1) tests of association are given for each previously reported SNP or its proxy in this African American dataset (n = 351). (DOCX) [file pone.0111301.s007.docx]

**Table S4: Comparison of associations in eMERGE African American TSH study participants to previously published SNP associations with serum TSH levels.** SNP rs number, chromosomal location, nearest gene/gene region, coded allele (CA), coded allele frequency (CAF), association summary statistics (betas, standard errors, and p-values), and PubMed ID (PMID) are given for each previously reported association with TSH levels in European Americans. CAF highlighted with (*) represents the average CAF in the Taylor et al. (PMID: 21317282) study. For SNPs not directly genotyped in this study, the proxy in highest linkage disequilibrium in 1000 Genomes CEU samples was identified. Results of adjusted (age, sex, BMI, and PC1) tests of association are given for each previously reported SNP or its proxy in this African American dataset (n = 351).

| **Locus** | | | **Prior Association** | | | | | **Current Study** | | | | | |
| --- | --- | --- | --- | --- | --- | --- | --- | --- | --- | --- | --- | --- | --- |
| **SNP** | **Chr** | **Gene** | **CA** | **CAF** | **β (SE)** | **P-value** | **PMID** | **SNP/Best Proxy SNP** | **r^2^** | **CA** | **CAF** | **β (SE)** | **P-value** |
|  |  |  |  |  |  |  |  |  |  |  |  |  |  |
| rs10917469 | 1 | *CAPZB* | G | 0.16 | -0.16 (0.03) | 3.2E-08 | 20826269 | rs12138950 | 1.00 | C | 0.24 | -0.02 (0.05) | 0.64 |
| rs10917477 | 1 | *CAPZB* | A | 0.51 | -0.06 (0.01) | 1.54E-08 | 22494929 | rs6683419 | 0.62 | G | 0.49 | 0.01 (0.04) | 0.85 |
| rs10799824 | 1 | *CAPZB* | A | 0.16 | -0.11 (0.01) | 3.60E-21 | 23408906 | rs10799824 | -- | A | 0.24 | -0.03 (0.05) | 0.58 |
| rs334699 | 1 | *NFIA* | A | 0.05 | -0.14 (0.02) | 5.40E-12 | 23408906 | rs334713 | 1.00 | A | 0.17 | -0.17 (0.05) | 1.50E-03 |
| rs13015993 | 2 | *IGFBP5* | A | 0.74 | 0.08 (0.01) | 3.24E-15 | 23408906 | rs13020935 | 1.00 | G | 0.48 | -0.15 (0.04) | 1.82E-04 |
| rs10028213 | 4 | *NR3C2* | C | 0.82 | 0.08 (0.01) | 2.88E-10 | 22494929 | rs10519980 | 1.00 | T | 0.33 | -0.07 (0.04) | 0.11 |
| rs10032216 | 4 | *NR3C2* | T | 0.78 | 0.09 (0.01) | 9.28E-16 | 23408906 | rs17025017 | 1.00 | A | 0.42 | -0.07 (0.04) | 0.08 |
| rs2046045 | 5 | *PDE8B* | T | 0.62 | -0.12 (0.01) | 2.79E-27 | 22494929 | rs2046045 | -- | A | 0.28 | -0.09 (0.04) | 0.03 |
| rs6885099 | 5 | *PDE8B* | A | 0.59 | -0.14 (0.01) | 1.95E-56 | 23408906 | rs2046045 | 1.00 | A | 0.28 | -0.09 (0.04) | 0.03 |
| rs4704397 | 5 | *PDE8B* | A | 0.41* | 0.21 | 1.64E-10 | 21367965 | rs2046045 | 0.94 | A | 0.28 | -0.09 (0.04) | 0.03 |
| rs753760 | 6 | *PDE10A* | C | 0.69 | 0.10 (0.01) | 1.21E-24 | 23408906 | rs2983514 | 0.93 | G | 0.38 | -0.01 (0.04) | 0.73 |
| rs9472138 | 6 | *VEGFA* | T | 0.29 | -0.08 (0.01) | 6.72E-16 | 23408906 | rs9472138 | -- | T | 0.19 | -0.10 (0.05) | 0.05 |
| rs11755845 | 6 | *VEGFA* | T | 0.27 | -0.07 (0.01) | 1.68E-10 | 23408906 | rs11755845 | -- | T | 0.14 | -0.13 (0.05) | 0.01 |
| rs9497965 | 6 | *SASH1* | T | 0.42 | 0.05 (0.01) | 2.25E-08 | 23408906 | rs9377117 | 0.54 | G | 0.18 | 0.01 (0.06) | 0.85 |
| rs7825175 | 8 | *NRG1* | A | 0.21 | -0.07 (0.01) | 2.94E-09 | 23408906 | rs7825175 | -- | A | 0.13 | -0.10 (0.06) | 0.12 |
| rs657152 | 9 | *ABO* | A | 0.34 | 0.06 (0.01) | 4.11E-10 | 23408906 | rs657152 | -- | T | 0.43 | 0.09 (0.04) | 0.03 |
| rs1571583 | 9 | *GLIS3* | A | 0.25 | 0.06 (0.01) | 2.55E-08 | 23408906 | rs1571583 | -- | T | 0.22 | 0.01 (0.05) | 0.79 |
| rs17723470 | 11 | *PRDM11* | T | 0.28 | -0.07 (0.01) | 8.83E-11 | 23408906 | rs17723470 | -- | T | 0.11 | -0.10 (0.06) | 0.11 |
| rs1537424 | 14 | *MBIP* | T | 0.61 | -0.05 (0.01) | 1.17E-08 | 23408906 | rs1537424 | -- | A | 0.34 | 0.04 (0.04) | 0.35 |
| rs11624776 | 14 | *ITPK1* | A | 0.66 | -0.06 (0.01) | 1.79E-09 | 23408906 | rs11624776 | -- | C | 0.11 | 0.04 (0.07) | 0.57 |
| rs10519227 | 15 | *FGF7* | A | 0.25 | -0.07 (0.01) | 1.02E-11 | 23408906 | rs7168316 | 1.00 | T | 0.12 | -0.03 (0.06) | 0.62 |
| rs17776563 | 15 | *MIR1179* | A | 0.32 | -0.06 (0.01) | 2.89E-10 | 23408906 | rs13329353 | 0.96 | C | 0.45 | -0.07 (0.04) | 0.09 |
| rs3813582 | 16 | LOC440389*/MAF* | T | 0.67 | 0.08 (0.01) | 8.45E-18 | 22494929, 23408906 | rs17767383 | 1.00 | A | 0.25 | -0.06 (0.05) | 0.18 |
| rs9915657 | 17 | *SOX9* | T | 0.54 | -0.06 (0.01) | 7.53E-13 | 23408906 | rs9915657 | -- | T | 0.49 | -0.06 (0.04) | 0.17 |
| rs4804416 | 19 | *INSR* | T | 0.57 | -0.06 (0.01) | 3.16E-10 | 23408906 | rs4804416 | -- | G | 0.26 | 0.01 (0.05) | 0.81 |

**References**

1. Panicker V, Wilson SG, Walsh JP, Richards JB, Brown SJ, Beilby JP, Bremner AP, Surdulescu GL, Qweitin E, Gillham-Nasenya I, Soranzo N, Lim EM, Fletcher SJ, Spector TD (2010) A locus on chromosome 1p36 is associated with thyrotropin and thyroid function as identified by genome-wide association study. Am J Hum Genet 87: 430-435. S0002-9297(10)00418-0 [pii];10.1016/j.ajhg.2010.08.005 [doi].

2. Rawal R, Teumer A, Volzke H, Wallaschofski H, Ittermann T, Asvold BO, Bjoro T, Greiser KH, Tiller D, Werdan K, Meyer Zu Schwabedissen HE, Doering A, Illig T, Gieger C, Meisinger C, Homuth G (2012) Meta-analysis of two genome-wide association studies identifies four genetic loci associated with thyroid function. Hum Mol Genet 21: 3275-3282. dds136 [pii];10.1093/hmg/dds136 [doi].

3. Medici M, van der Deure WM, Verbiest M, Vermeulen SH, Hansen PS, Kiemeney LA, Hermus AR, Breteler MM, Hofman A, Hegedus L, Kyvik KO, den HM, Uitterlinden AG, Visser TJ, Peeters RP (2011) A large-scale association analysis of 68 thyroid hormone pathway genes with serum TSH and FT4 levels. Eur J Endocrinol 164: 781-788. EJE-10-1130 [pii];10.1530/EJE-10-1130 [doi].
