# Supplementary material for: Genetic Variants Associated with Serum Thyroid Stimulating Hormone (TSH) Levels in European Americans and African Americans from the eMERGE Network
Source: PLoS One. 2014 Dec 1;9(12):e111301. doi: 10.1371/journal.pone.0111301 (PMC4249871; doi:10.1371/journal.pone.0111301)
Supplement: Table S6 — Comparison of associations in eMERGE African Americans with previously published SNP associations for thyroid-related traits. SNP rs number, chromosomal location, nearest gene/gene region, coded allele (CA), coded allele frequency (CAF), and association summary statistics (odds ratio (OR) and p-values) are given for each previously reported association with thyroid-related traits in European Americans. For SNPs not directly genotyped in this study, the proxy in highest linkage disequilibrium in 1000 Genomes CEU samples was identified. Results of adjusted (age, sex, body mass index, and principal component 1) tests of association are given for each previously reported SNP or its proxy in this African American dataset (n = 351). (DOCX) [file pone.0111301.s009.docx]

**Table S6: Comparison of associations in eMERGE African Americans with previously published SNP associations for thyroid-related traits.** SNP rs number, chromosomal location, nearest gene/gene region, coded allele (CA), coded allele frequency (CAF), and association summary statistics (odds ratio (OR) and p-values) are given for each previously reported association with thyroid-related traits in European Americans. For SNPs not directly genotyped in this study, the proxy in highest linkage disequilibrium in 1000 Genomes CEU samples was identified. Results of adjusted (age, sex, body mass index, and principal component 1) tests of association are given for each previously reported SNP or its proxy in this African American dataset (n = 351).

| **Locus** | | | | | **Prior Association** | | | | | | | | | | | | | **Current Study** | | | | | | | | | | | | | |
| --- | --- | --- | --- | --- | --- | --- | --- | --- | --- | --- | --- | --- | --- | --- | --- | --- | --- | --- | --- | --- | --- | --- | --- | --- | --- | --- | --- | --- | --- | --- | --- |
| **SNP** | **Chr** | | **Gene** | | **CA** | **CAF** | | | **OR** | **P-value** | | | | | **Ref.** | | | **SNP/Best Proxy SNP** | | | **r^2^** | **CA** | | | **CAF** | | | | **Β (SE)** | **P-value** | |
| **Hypothyroidism** | | | | | | | | | | | | | | | | | | | | | | | | | | | | | | | |
| rs6679677 | 1 | | *PHTF1, RSBN1* | | A | 0.09 | | | 1.36 | 2.80E-13 | | | | | 22493691 | | | rs1217413 | | | 0.60 | G | | | 0.05 | | | | 0.08  (0.10) | 0.43 | |
| rs2476601 | 1 | | *PTPN22* | | A | 0.09 | | | 1.36 | 3.9E-13 | | | | | 22493691 | | | rs1217413 | | | 0.56 | G | | | 0.05 | | | | 0.08  (0.10) | 0.43 | |
| rs4915076 | 1 | | *VAV3* | | C | 0.08 | | | 1.3 | 8.00E-10 | | | | | 22493691 | | | rs4915076 | | | -- | C | | | 0.05 | | | | -0.03  (0.08) | 0.72 | |
| rs2517532 | 6 | | *LOC729792 HCG22* | | A | 0.40 | | | 0.86 | 1.3E-08 | | | | | 22493691 | | | rs2517532 | | | -- | T | | | 0.34 | | | | -0.02  (0.04) | 0.59 | |
| rs1064191 | 6 | | *HCG22/C6orf15* | | T | 0.46 | | | 0.87 | 2.2E-08 | | | | | 22493691 | | | rs1064191 | | | -- | A | | | 0.46 | | | | -0.04  (0.04) | 0.36 | |
| rs925487 | 9 | | *FOXE1/C9orf156* | | C | 0.37 | | | 0.86 | 4.1E-08 | | | | | 22493691 | | | rs925487 | | | -- | G | | | 0.25 | | | | -0.04  (0.05) | 0.41 | |
| rs907580 | 9 | | *FOXE1/C9orf156* | | T | 0.26 | | | 0.84 | 1.2E-08 | | | | | 22493691 | | | rs907580 | | | -- | A | | | 0.07 | | | | 0.04  (0.08) | 0.64 | |
| rs925489 | 9 | | *KRT18P13,FOXE1* | | C | 0.33 | | | 0.78 | 2.40E-19 | | | | | 22493691 | | | rs925489 | | | -- | C | | | 0.21 | | | | -0.03  (0.05) | 0.53 | |
| rs1877432 | 9 | | *KRT18P13,FOXE1* | | A | 0.40 | | | 1.16 | 4.40E-09 | | | | | 22493691 | | | rs1877432 | | | -- | A | | | 0.34 | | | | 0.11  (0.04) | 9.73E-03 | |
| rs7024345 | 9 | | *KRT18P13,FOXE1* | | A | 0.26 | | | 0.84 | 1E-08 | | | | | 22493691 | | | rs7024345 | | | -- | A | | | 0.07 | | | | 0.03  (0.08) | 0.69 | |
| rs7848973 | 9 | | *KRT18P13,FOXE1* | | A | 0.40 | | | 0.84 | 7.10E-11 | | | | | 22493691 | | | rs7848973 | | | -- | A | | | 0.23 | | | | -0.09  (0.04) | 0.06 | |
| rs11065987 | 12 | | *LOC100101246 BRAP* | | G | 0.45 | | | 1.18 | 1.70E-10 | | | | | 22493691 | | | rs11065987 | | | -- | G | | | 0.08 | | | | 0.12  (0.08) | 0.13 | |
| rs17696736 | 12 | | *NAA25* | | G | 0.46 | | | 1.18 | 2.80E-10 | | | | | 22493691 | | | rs17696736 | | | -- | G | | | 0.09 | | | | 0.11  (0.08) | 0.16 | |
| rs11066320 | 12 | | *PTPN11* | | A | 0.45 | | | 1.17 | 3.50E-09 | | | | | 22493691 | | | rs11066320 | | | -- | A | | | 0.08 | | | | 0.08  (0.08) | 0.31 | |
| rs3184504 | 12 | | *SH2B3* | | C | 0.50 | | | 0.84 | 2.60E-12 | | | | | 22493691 | | | rs3184504 | | | -- | T | | | 0.10 | | | | 0.09  (0.07) | 0.20 | |
| rs11066188 | 12 | | *C12orf51* | | A | 0.44 | | | 1.18 | 4.1E-10 | | | | | 22493691 | | | rs11066188 | | | -- | A | | | 0.08 | | | | 0.12  (0.08) | 0.12 | |
| rs653178 | 12 | | *ATXN2* | | T | 0.50 | | | 0.84 | 5.0E-12 | | | | | 22493691 | | | rs653178 | | | -- | G | | | 0.10 | | | | 0.09  (0.07) | 0.20 | |
|  | | | | | | | | | | | | | | | | | | | | | | | | | | | | | | | |
| **Grave’s Disease/Autoimmune Thyroid Disease** | | | | | | | | | | | | | | | | | | | | | | | | | | | | | | | |
| rs3761959 | 1 | | *FCRL3* | | A | | 0.40 | 1.23 | | | | 1.50E-13 | | 21841780 | | | rs3761959 | | | -- | | | | G | | 0.39 | | 0.03  (0.04) | | 0.45 | |
| rs1024161 | 2 | | *CTLA4* | | T | | 0.69 | 1.3 | | | | 2.34E-17 | | 21841780 | | | rs1024161 | | | -- | | | | C | | 0.48 | | -0.06  (0.09) | | 0.13 | |
| rs6832151 | 4 | | *RHOH,CHRNA9* | | G | | 0.35 | 1.24 | | | | 1.08E-13 | | 21841780 | | | rs6832151 | | | -- | | | | G | | 0.31 | | -0.10  (0.04) | | 0.01 | |
| rs9355610 | 6 | | *RNASET2* | | G | | 0.47 | 1.19 | | | | 6.85E-10 | | 21841780 | | | rs9355610 | | | -- | | | | A | | 0.39 | | -0.03  (0.04) | | 0.47 | |
| rs4947296 | 6 | | *MUC21,C6orf15* | | C | | 0.14 | 1.77 | | | | 3.51E-51 | | 21841780 | | | NA | | | NA | | | | NA | | NA | | NA | | NA | |
| rs2281388 | 6 | | *HLA-DPB1* | | T | | 0.32 | 1.64 | | | | 1.5E-65 | | 21841780 | | | NA | | | NA | | | | NA | | NA | | NA | | NA | |
| rs6457617 | 6 | | *HLA-DR-DQ* | | T | | 0.45 | 1.4 | | | | 7.38E-33 | | 21841780 | | | rs6457617 | | | -- | | | | T | | 0.48 | | 0.02  (0.04) | | 0.63 | |
| rs6903608 | 6 | | *HLA-DR-DQ* | | C | | 0.38 | 1.34 | | | | 5.12E-24 | | 21841780 | | | rs6903608 | | | -- | | | | C | | 0.38 | | 0.02  (0.04) | | 0.61 | |
| rs965513 | 9 | | *FOXE1* | | A | | 0.34 | 1.75 | | | | 1.70E-27 | | 22493691 | | | rs965513 | | | -- | | | | A | | 0.17 | | -0.03  (0.05) | | 0.50 | |
| rs12101261 | 14 | | *TSHR* | | T | | 0.64 | 1.35 | | | | 6.64E-24 | | 21841780 | | | rs12101261 | | | -- | | | | T | | 0.39 | | -0.01  (0.04) | | 0.82 | |
|  | | | | | | | | | | | | | | | | | | | | | | | | | | | | | | | |
| **Thyroid Cancer** | | | | | | | | | | | | | | | | | | | | | | | | | | | | | | | |
| rs966423 | | 2 | *DIRC3* | C | | |  | 1.34 | | | 1.30E-09 | | 22267200 | | | rs966423 | | | -- | | | | T | | 0.23 | | 0.03  (0.05) | | | | 0.47 |
| rs2439302 | | 8 | *NRG1* | G | | |  | 1.36 | | | 2.00E-09 | | 22267200 | | | rs4733130 | | | 1.00 | | | | C | | 0.23 | | -0.06  (0.05) | | | | 0.25 |
| rs944289 | | 14 | *NKX2-1/TTF1* | T | | | 0.57 | 1.37 | | | 2E-09 | | 22267200, 19198613 | | | rs1169151 | | | 0.93 | | | | A | | 0.23 | | 0.03  (0.05) | | | | 0.57 |
| rs116909374 | | 14 | *MBIP* | T | | |  | 2.09 | | | 4.60E-11 | | 22267200 | | | NA | | | NA | | | | NA | | NA | | NA | | | | NA |

**References**

1. Eriksson N, Tung JY, Kiefer AK, Hinds DA, Francke U, Mountain JL, Do CB (2012) Novel associations for hypothyroidism include known autoimmune risk loci. PLoS One 7: e34442. 10.1371/journal.pone.0034442 [doi];PONE-D-11-14352 [pii].

2. Chu X, Pan CM, Zhao SX, Liang J, Gao GQ, Zhang XM, Yuan GY, Li CG, Xue LQ, Shen M, Liu W, Xie F, Yang SY, Wang HF, Shi JY, Sun WW, Du WH, Zuo CL, Shi JX, Liu BL, Guo CC, Zhan M, Gu ZH, Zhang XN, Sun F, Wang ZQ, Song ZY, Zou CY, Sun WH, Guo T, Cao HM, Ma JH, Han B, Li P, Jiang H, Huang QH, Liang L, Liu LB, Chen G, Su Q, Peng YD, Zhao JJ, Ning G, Chen Z, Chen JL, Chen SJ, Huang W, Song HD (2011) A genome-wide association study identifies two new risk loci for Graves' disease. Nat Genet 43: 897-901. ng.898 [pii];10.1038/ng.898 [doi].

3. Gudmundsson J, Sulem P, Gudbjartsson DF, Jonasson JG, Masson G, He H, Jonasdottir A, Sigurdsson A, Stacey SN, Johannsdottir H, Helgadottir HT, Li W, Nagy R, Ringel MD, Kloos RT, de Visser MC, Plantinga TS, den HM, Aguillo E, Panadero A, Prats E, Garcia-Castano A, De JA, Rivera F, Walters GB, Bjarnason H, Tryggvadottir L, Eyjolfsson GI, Bjornsdottir US, Holm H, Olafsson I, Kristjansson K, Kristvinsson H, Magnusson OT, Thorleifsson G, Gulcher JR, Kong A, Kiemeney LA, Jonsson T, Hjartarson H, Mayordomo JI, Netea-Maier RT, de la Chapelle A, Hrafnkelsson J, Thorsteinsdottir U, Rafnar T, Stefansson K (2012) Discovery of common variants associated with low TSH levels and thyroid cancer risk. Nat Genet 44: 319-322. ng.1046 [pii];10.1038/ng.1046 [doi].

4. Gudmundsson J, Sulem P, Gudbjartsson DF, Jonasson JG, Sigurdsson A, Bergthorsson JT, He H, Blondal T, Geller F, Jakobsdottir M, Magnusdottir DN, Matthiasdottir S, Stacey SN, Skarphedinsson OB, Helgadottir H, Li W, Nagy R, Aguillo E, Faure E, Prats E, Saez B, Martinez M, Eyjolfsson GI, Bjornsdottir US, Holm H, Kristjansson K, Frigge ML, Kristvinsson H, Gulcher JR, Jonsson T, Rafnar T, Hjartarsson H, Mayordomo JI, de la Chapelle A, Hrafnkelsson J, Thorsteinsdottir U, Kong A, Stefansson K (2009) Common variants on 9q22.33 and 14q13.3 predispose to thyroid cancer in European populations. Nat Genet 41: 460-464. ng.339 [pii];10.1038/ng.339 [doi].
