# Supplementary material for: Genetic Variants Associated with Serum Thyroid Stimulating Hormone (TSH) Levels in European Americans and African Americans from the eMERGE Network
Source: PLoS One. 2014 Dec 1;9(12):e111301. doi: 10.1371/journal.pone.0111301 (PMC4249871; doi:10.1371/journal.pone.0111301)
Supplement: Table S7 — Comparison of SNP associations (p<10−04) in regression models with and without body mass index covariates for serum TSH levels in euthyroid eMERGE study European Americans (n = 4,501). For each SNP, p-values and betas are given for models that include or exclude BMI as a covariate. All models are linear regressions assuming an additive genetic model adjusted for age, sex, and principal component 1. (DOCX) [file pone.0111301.s010.docx]

**Table S7. Comparison of SNP associations (p<10^-04^) in regression models with and without body mass index covariates for serum TSH levels in euthyroid eMERGE study European Americans (n=4,501).** For each SNP, p-values and betas are given for models that include or exclude BMI as a covariate. All models are linear regressions assuming an additive genetic model adjusted for age, sex, and principal component 1.

| **SNP** | **P BMI** | **BETA BMI** | **P NO BMI** | **BETA NO BMI** |
| --- | --- | --- | --- | --- |
| rs1382879 | 7.16E-18 | 0.08707 | 2.162E-15 | 0.07515 |
| rs2046045 | 1.85E-17 | 0.08588 | 2.071E-15 | 0.07512 |
| rs989758 | 1.33E-14 | 0.07929 | 1.096E-12 | 0.06853 |
| rs9687206 | 5.52E-14 | 0.07513 | 4.976E-11 | 0.06141 |
| rs12515498 | 3.27E-10 | 0.0709 | 3.177E-09 | 0.0626 |
| rs6885813 | 4.05E-08 | 0.06293 | 1.416E-07 | 0.05619 |
| rs1096752 | 6.30E-07 | -0.04902 | 7.483E-06 | -0.04132 |
| rs13361710 | 6.60E-07 | 0.05748 | 0.0000022 | 0.05089 |
| rs10759944 | 1.08E-06 | -0.05123 | 0.00000133 | -0.04751 |
| rs965513 | 1.09E-06 | -0.05108 | 1.395E-06 | -0.04732 |
| rs925489 | 1.79E-06 | -0.05009 | 2.521E-06 | -0.04619 |
| rs7850258 | 1.85E-06 | -0.05003 | 2.869E-06 | -0.04594 |
| rs10496992 | 2.22E-06 | 0.04843 | 0.00001611 | 0.04137 |
| rs1861628 | 3.68E-06 | -0.05088 | 0.00002153 | -0.0437 |
| rs4348174 | 3.97E-06 | 0.04619 | 0.00001128 | 0.04111 |
| rs657152 | 4.18E-06 | 0.04659 | 1.063E-07 | 0.05058 |
| rs740083 | 4.56E-06 | -0.05314 | 0.00001372 | -0.04737 |
| rs813379 | 4.57E-06 | -0.0995 | 2.661E-06 | -0.09506 |
| rs2712168 | 4.98E-06 | 0.06736 | 0.00002046 | 0.05825 |
| rs256438 | 5.53E-06 | 0.04675 | 0.00001808 | 0.04128 |
| rs4570936 | 5.73E-06 | -0.05379 | 2.324E-07 | -0.05758 |
| rs6546537 | 5.92E-06 | -0.04988 | 0.00002922 | -0.04312 |
| rs7855088 | 6.23E-06 | -0.04507 | 0.0000237 | -0.03953 |
| rs925487 | 6.24E-06 | -0.04622 | 6.994E-06 | -0.04315 |
| rs803174 | 6.74E-06 | -0.09764 | 3.383E-06 | -0.09407 |
| rs2438632 | 6.88E-06 | 0.04561 | 0.00001008 | 0.04202 |
| rs13020935 | 7.02E-06 | -0.04906 | 0.00003453 | -0.04234 |
| rs12520862 | 7.48E-06 | 0.06316 | 0.00009363 | 0.0516 |
| rs10984103 | 7.81E-06 | -0.04571 | 7.692E-06 | -0.04293 |
| rs907580 | 8.20E-06 | -0.04946 | 6.144E-06 | -0.04705 |
| rs2466067 | 8.42E-06 | -0.04676 | 5.858E-07 | -0.04924 |
| rs7870926 | 8.67E-06 | -0.04423 | 0.00001468 | -0.04039 |
| rs7341064 | 1.03E-05 | 0.04421 | 0.00002182 | 0.0398 |
| rs4298457 | 1.07E-05 | -0.04863 | 1.045E-06 | -0.05055 |
| rs598599 | 1.09E-05 | 0.04815 | 0.0000883 | 0.04028 |
| rs4693596 | 1.10E-05 | -0.04467 | 0.0001054 | -0.0372 |
| rs10954859 | 1.12E-05 | -0.04833 | 0.00000121 | -0.05009 |
| rs404375 | 1.26E-05 | -0.04282 | 0.00002377 | -0.03892 |
| rs2983525 | 1.34E-05 | -0.04789 | 0.00000104 | -0.05046 |
| rs2983514 | 1.36E-05 | -0.04554 | 6.447E-07 | -0.04897 |
| rs3766122 | 1.42E-05 | -0.09985 | 0.0007203 | -0.07111 |
| rs7866436 | 1.52E-05 | -0.04427 | 0.00001553 | -0.04146 |
| rs7024345 | 1.65E-05 | -0.04755 | 0.00001406 | -0.04503 |
| rs26367 | 1.65E-05 | -0.07248 | 0.00002052 | -0.06721 |
| rs10073636 | 1.73E-05 | 0.04274 | 0.0001353 | 0.03561 |
| rs13285674 | 1.90E-05 | 0.04926 | 0.00001864 | 0.04618 |
| rs505922 | 1.94E-05 | 0.04384 | 3.549E-07 | 0.0492 |
| rs7445986 | 1.95E-05 | 0.04293 | 0.0000584 | 0.0378 |
| rs10204522 | 1.95E-05 | 0.07346 | 0.00003885 | 0.06506 |
| rs4054489 | 1.95E-05 | -0.05422 | 0.00007092 | -0.04757 |
| rs1055075 | 1.96E-05 | -0.04469 | 0.00001442 | -0.04259 |
| rs4861534 | 2.09E-05 | 0.0674 | 0.00002146 | 0.0634 |
| rs7168316 | 2.10E-05 | -0.0499 | 0.0000255 | -0.04661 |
| rs7848973 | 2.11E-05 | -0.04248 | 0.0000222 | -0.03973 |
| rs3136559 | 2.13E-05 | 0.04623 | 0.0008506 | 0.03415 |
| rs6727435 | 2.15E-05 | -0.047 | 0.00009244 | -0.04055 |
| rs33613 | 2.35E-05 | -0.07251 | 0.00002682 | -0.06739 |
| rs12592277 | 2.35E-05 | -0.05029 | 0.00002394 | -0.04737 |
| rs2466062 | 2.36E-05 | -0.04528 | 0.0000015 | -0.04831 |
| rs3898456 | 2.56E-05 | 0.04331 | 0.00009802 | 0.03746 |
| rs4402960 | 2.63E-05 | -0.04536 | 0.00001032 | -0.04452 |
| rs1470579 | 2.67E-05 | -0.04518 | 8.352E-06 | -0.04484 |
| rs13354798 | 2.75E-05 | 0.04172 | 0.0001914 | 0.03484 |
| rs9686502 | 2.85E-05 | 0.04109 | 0.0003828 | 0.03265 |
| rs9606756 | 2.86E-05 | 0.06595 | 0.0001274 | 0.05696 |
| rs494442 | 3.03E-05 | -0.04159 | 0.0001762 | -0.03509 |
| rs2695148 | 3.11E-05 | -0.06891 | 0.00009136 | -0.06032 |
| rs17265852 | 3.16E-05 | -0.07471 | 0.00003343 | -0.06941 |
| rs6414906 | 3.51E-05 | 0.04134 | 0.0001805 | 0.03511 |
| rs3813583 | 4.06E-05 | 0.04111 | 5.944E-06 | 0.04253 |
| rs749378 | 4.09E-05 | -0.04505 | 0.000103 | -0.04 |
| rs6451801 | 4.10E-05 | 0.04102 | 0.0002265 | 0.03461 |
| rs13162651 | 4.11E-05 | 0.041 | 0.0002136 | 0.03474 |
| rs12201217 | 4.30E-05 | -0.04117 | 0.00001856 | -0.04042 |
| rs370234 | 4.32E-05 | -0.04073 | 0.00006801 | -0.03727 |
| rs1647253 | 4.57E-05 | -0.06761 | 0.0001226 | -0.05932 |
| rs6989877 | 4.59E-05 | 0.0592 | 3.061E-06 | 0.06399 |
| rs11963665 | 4.63E-05 | -0.0494 | 0.0002728 | -0.0417 |
| rs6892290 | 4.76E-05 | 0.04069 | 0.0002405 | 0.03448 |
| rs6668505 | 4.88E-05 | -0.0825 | 0.0001846 | -0.07155 |
| rs3745746 | 4.93E-05 | -0.04072 | 0.00008119 | -0.03703 |
| rs12521494 | 5.00E-05 | 0.04685 | 0.001045 | 0.03538 |
| rs10064949 | 5.07E-05 | 0.03991 | 0.00007765 | 0.03647 |
| rs1515259 | 5.44E-05 | 0.04 | 0.0003208 | 0.03339 |
| rs1012319 | 5.47E-05 | -0.05013 | 0.0001041 | -0.04521 |
| rs2983500 | 5.54E-05 | -0.06371 | 4.798E-06 | -0.06777 |
| rs8096947 | 5.56E-05 | -0.05073 | 0.00000939 | -0.0524 |
| rs888186 | 5.63E-05 | -0.0675 | 0.0002033 | -0.05867 |
| rs4703797 | 5.65E-05 | 0.0423 | 0.00007767 | 0.03885 |
| rs529126 | 5.65E-05 | 0.04482 | 0.0001849 | 0.03912 |
| rs11805172 | 5.69E-05 | -0.0771 | 0.0002088 | -0.06707 |
| rs8009673 | 5.83E-05 | 0.05526 | 0.00003141 | 0.05331 |
| rs1939422 | 5.94E-05 | -0.04114 | 0.0002349 | -0.03543 |
| rs1986415 | 6.05E-05 | 0.06231 | 0.0001559 | 0.05442 |
| rs2439300 | 6.19E-05 | -0.04402 | 0.00001906 | -0.04405 |
| rs2943179 | 6.31E-05 | 0.04715 | 0.00004182 | 0.04541 |
| rs8035662 | 6.37E-05 | -0.04129 | 0.0002975 | -0.03483 |
| rs877138 | 6.37E-05 | -0.04171 | 0.00004111 | -0.04007 |
| rs1443434 | 6.53E-05 | -0.04033 | 0.00005971 | -0.03806 |
| rs2381866 | 6.68E-05 | 0.03978 | 0.00009972 | 0.0364 |
| rs888182 | 6.75E-05 | 0.05466 | 0.00003552 | 0.05257 |
| rs7184757 | 7.22E-05 | -0.07004 | 0.00006698 | -0.06502 |
| rs11172482 | 7.29E-05 | -0.04016 | 0.0001113 | -0.03674 |
| rs39334 | 7.47E-05 | 0.0406 | 0.0002377 | 0.03539 |
| rs12278001 | 7.53E-05 | -0.08195 | 0.00008236 | -0.07641 |
| rs12654213 | 7.69E-05 | 0.03922 | 0.0003638 | 0.03318 |
| rs10489909 | 7.81E-05 | -0.0875 | 0.0008428 | -0.06914 |
| rs13231383 | 8.19E-05 | 0.04496 | 0.0001714 | 0.04017 |
| rs2306344 | 8.23E-05 | -0.04227 | 0.00006537 | -0.04001 |
| rs11666426 | 8.30E-05 | 0.03938 | 0.00008649 | 0.03686 |
| rs12138950 | 8.97E-05 | -0.05446 | 0.0001294 | -0.04953 |
| rs424829 | 9.02E-05 | 0.0428 | 0.0003391 | 0.03675 |
| rs11071858 | 9.33E-05 | -0.03879 | 0.0004399 | -0.03252 |
| rs12282135 | 9.47E-05 | -0.05473 | 0.0002436 | -0.04844 |
| rs11118832 | 9.52E-05 | -0.07324 | 0.0001705 | -0.06547 |
| rs630505 | 9.58E-05 | -0.0435 | 0.0001384 | -0.03995 |
| rs16856529 | 9.80E-05 | 0.05392 | 0.000059 | 0.05159 |
| rs1502816 | 9.95E-05 | -0.0393 | 0.0001324 | -0.03624 |
