# Supplementary material for: Genetic Variants Associated with Serum Thyroid Stimulating Hormone (TSH) Levels in European Americans and African Americans from the eMERGE Network
Source: PLoS One. 2014 Dec 1;9(12):e111301. doi: 10.1371/journal.pone.0111301 (PMC4249871; doi:10.1371/journal.pone.0111301)
Supplement: Table S8 — Comparison of SNP associations (p<10−04) in regression models with and without body mass index covariates for serum TSH levels in euthyroid eMERGE study African Americans (n = 351). For each SNP, p-values and betas are given for models that include or exclude BMI as a covariate. All models are linear regressions assuming an additive genetic model adjusted for age, sex, and principal component 1. (DOCX) [file pone.0111301.s011.docx]

**Table S8: Comparison of SNP associations (p<10^-04^) in regression models with and without body mass index covariates for serum TSH levels in euthyroid eMERGE study African Americans (n=351).** For each SNP, p-values and betas are given for models that include or exclude BMI as a covariate. All models are linear regressions assuming an additive genetic model adjusted for age, sex, and principal component 1.

| **SNP** | **P BMI** | **BETA BMI** | **P NO BMI** | **BETA NO BMI** |
| --- | --- | --- | --- | --- |
| rs1409005 | 5.02E-07 | 0.25 | 0.000026 | 0.17 |
| rs2378497 | 3.53E-06 | 0.33 | 0.004762 | 0.16 |
| rs6062344 | 4.06E-06 | 0.18 | 0.003641 | 0.09 |
| rs270421 | 7.75E-06 | 0.19 | 0.003076 | 0.10 |
| rs2299116 | 8.16E-06 | 0.25 | 6.13E-05 | 0.18 |
| rs6728613 | 1.14E-05 | 0.20 | 0.007952 | 0.10 |
| rs6585018 | 1.17E-05 | -0.22 | 0.000258 | -0.14 |
| rs1013757 | 1.33E-05 | -0.19 | 0.000732 | -0.12 |
| rs4073401 | 1.33E-05 | 0.19 | 0.00828 | 0.09 |
| rs12883861 | 1.63E-05 | 0.21 | 0.000222 | 0.14 |
| rs9784959 | 1.82E-05 | -0.18 | 1.63E-05 | -0.15 |
| rs270422 | 2.17E-05 | 0.18 | 0.01112 | 0.09 |
| rs261875 | 2.24E-05 | 0.18 | 3.56E-05 | 0.14 |
| rs274614 | 2.36E-05 | -0.18 | 0.025 | -0.08 |
| rs11711934 | 2.45E-05 | -0.17 | 0.001414 | -0.11 |
| rs12621889 | 2.68E-05 | 0.36 | 0.01186 | 0.16 |
| rs12464144 | 2.68E-05 | 0.36 | 0.01186 | 0.16 |
| rs10163845 | 2.74E-05 | -0.18 | 0.04236 | -0.07 |
| rs12610504 | 3.07E-05 | 0.20 | 0.00244 | 0.12 |
| rs1274744 | 3.21E-05 | -0.17 | 0.001977 | -0.10 |
| rs10060607 | 3.28E-05 | 0.18 | 8.25E-05 | 0.13 |
| rs1824304 | 3.32E-05 | 0.17 | 0.000922 | 0.11 |
| rs841452 | 3.52E-05 | 0.17 | 0.001955 | 0.10 |
| rs11977108 | 3.70E-05 | -0.21 | 0.00062 | -0.14 |
| rs4678798 | 3.71E-05 | 0.24 | 8E-05 | 0.18 |
| rs6851816 | 3.83E-05 | 0.16 | 0.00065 | 0.10 |
| rs133201 | 4.04E-05 | 0.27 | 0.0513 | 0.11 |
| rs2593996 | 4.09E-05 | -0.16 | 5.52E-05 | -0.13 |
| rs1054713 | 4.16E-05 | 0.19 | 0.009705 | 0.10 |
| rs12609319 | 4.23E-05 | 0.20 | 0.002757 | 0.12 |
| rs1918092 | 4.90E-05 | 0.30 | 0.000447 | 0.20 |
| rs2303478 | 5.11E-05 | 0.18 | 0.02221 | 0.08 |
| rs3738605 | 5.12E-05 | 0.24 | 0.00633 | 0.13 |
| rs2659099 | 5.13E-05 | 0.18 | 0.006376 | 0.10 |
| rs4955261 | 5.19E-05 | 0.16 | 0.000377 | 0.11 |
| rs4772145 | 5.23E-05 | 0.15 | 0.02881 | 0.07 |
| rs13403407 | 5.31E-05 | -0.16 | 0.004395 | -0.09 |
| rs1513476 | 5.46E-05 | 0.22 | 0.000117 | 0.17 |
| rs17032566 | 5.52E-05 | -0.30 | 0.00145 | -0.19 |
| rs1105813 | 5.61E-05 | 0.16 | 0.001066 | 0.10 |
| rs1563333 | 5.64E-05 | -0.19 | 0.00206 | -0.11 |
| rs1907356 | 5.71E-05 | -0.21 | 0.000226 | -0.15 |
| rs11001788 | 5.71E-05 | -0.21 | 0.000226 | -0.15 |
| rs12470895 | 5.86E-05 | 0.21 | 0.001462 | 0.13 |
| rs646929 | 5.96E-05 | 0.30 | 0.000719 | 0.19 |
| rs2335640 | 5.98E-05 | -0.17 | 0.003313 | -0.10 |
| rs3742049 | 6.08E-05 | 0.18 | 0.003307 | 0.11 |
| rs17052068 | 6.46E-05 | -0.16 | 0.001956 | -0.10 |
| rs2819757 | 6.49E-05 | 0.22 | 0.003768 | 0.13 |
| rs10804139 | 6.54E-05 | -0.16 | 0.000886 | -0.11 |
| rs736218 | 6.66E-05 | 0.16 | 0.002017 | 0.10 |
| rs10989120 | 7.05E-05 | -0.19 | 0.1072 | -0.06 |
| rs10744020 | 7.05E-05 | 0.16 | 0.001966 | 0.10 |
| rs2659103 | 7.29E-05 | 0.19 | 0.01238 | 0.10 |
| rs10918914 | 7.39E-05 | 0.22 | 0.000129 | 0.16 |
| rs261878 | 7.42E-05 | -0.16 | 7.12E-05 | -0.13 |
| rs12914266 | 7.58E-05 | 0.17 | 0.001164 | 0.11 |
| rs6965055 | 7.65E-05 | -0.16 | 0.001335 | -0.10 |
| rs7808606 | 7.66E-05 | -0.15 | 0.000937 | -0.10 |
| rs17322359 | 7.74E-05 | 0.25 | 0.004053 | 0.15 |
| rs11949641 | 7.89E-05 | 0.18 | 0.00212 | 0.11 |
| rs12120382 | 7.96E-05 | 0.29 | 0.000834 | 0.20 |
| rs6731363 | 7.99E-05 | 0.20 | 0.001768 | 0.13 |
| rs13144021 | 8.00E-05 | 0.23 | 0.001055 | 0.15 |
| rs877128 | 8.10E-05 | 0.18 | 0.002341 | 0.11 |
| rs7923004 | 8.19E-05 | -0.20 | 0.001029 | -0.13 |
| rs6999969 | 8.33E-05 | -0.16 | 0.007186 | -0.09 |
| rs1027388 | 8.36E-05 | -0.17 | 0.002 | -0.11 |
| rs17011253 | 8.38E-05 | 0.27 | 0.03197 | 0.12 |
| rs942077 | 8.47E-05 | -0.15 | 0.000158 | -0.12 |
| rs4370216 | 8.55E-05 | -0.15 | 0.0018 | -0.10 |
| rs2333727 | 8.55E-05 | -0.15 | 0.0018 | -0.10 |
| rs1029357 | 8.57E-05 | 0.15 | 0.001052 | 0.10 |
| rs1332598 | 8.69E-05 | -0.19 | 0.1111 | -0.06 |
| rs6864667 | 8.98E-05 | 0.15 | 0.002212 | 0.09 |
| rs4411338 | 9.05E-05 | 0.16 | 0.01921 | 0.08 |
| rs171953 | 9.07E-05 | -0.15 | 0.02878 | -0.07 |
| GA035020 | 9.11E-05 | 0.19 | 0.01349 | 0.10 |
| rs2040578 | 9.13E-05 | 0.17 | 0.000272 | 0.13 |
| rs1106826 | 9.23E-05 | 0.17 | 0.01256 | 0.08 |
| rs6090040 | 9.35E-05 | 0.15 | 0.01731 | 0.08 |
| rs4408777 | 9.39E-05 | 0.16 | 0.06607 | 0.06 |
| rs2521676 | 9.73E-05 | 0.16 | 0.00084 | 0.11 |
| rs16845412 | 9.76E-05 | 0.27 | 0.006135 | 0.15 |
| rs10518306 | 9.78E-05 | 0.35 | 0.004332 | 0.19 |
| rs10098991 | 9.86E-05 | 0.16 | 0.07677 | 0.06 |
| rs8059691 | 9.90E-05 | 0.23 | 0.01467 | 0.11 |
