# Supplementary material for: Genetic Variants Associated with Serum Thyroid Stimulating Hormone (TSH) Levels in European Americans and African Americans from the eMERGE Network
Source: PLoS One. 2014 Dec 1;9(12):e111301. doi: 10.1371/journal.pone.0111301 (PMC4249871; doi:10.1371/journal.pone.0111301)
Supplement: Table S9 — Body mass index as a modifier of serum TSH levels genetic associations. Interaction analyses were performed using the SNPs with p<1×10^−04^ significance levels in the model adjusted for age, sex, principal component (PC) 1, and BMI in African Americans (n = 351); the model was stratified by race/ethnicity and by normal/overweight BMI (normal: BMI 18–24.9; overweight: BMI 25+). We considered a SNPxBMI interaction significant at a threshold of p<0.05. Displayed are significant interaction results at p = 0.05. (DOCX) [file pone.0111301.s012.docx]

**Table S9: Body mass index as a modifier of serum TSH levels genetic associations.** Interaction analyses were performed using the SNPs with p<1x10^-04^ significance levels in the model adjusted for age, sex, principal component (PC) 1, and BMI in African Americans (n=351); the model was stratified by race/ethnicity and by normal/overweight BMI (normal: BMI 18-24.9; overweight: BMI 25+). We considered a SNPxBMI interaction significant at a threshold of p<0.05. Displayed are significant interaction results at p=0.05.

| **POPULATION** | **SNP** | **GENE/REGION** | **MODIFIER** | **BETA (SE SNPxBMI)** | **P (SNPxBMI)** |
| --- | --- | --- | --- | --- | --- |
| European American | rs10489909 | *NFIA* | BMI | 0.01(0.004) | 6.21E-03 |
| European American | rs2466067 | *NRG1* | BMI | 0.004(0.002) | 0.040 |
| European American | rs4298457 | *NRG1* | BMI | 0.004(0.002) | 0.047 |
| European American | rs10954859 | *NRG1* | BMI | 0.004(0.002) | 0.050 |
| African American | rs6728613 | *MYT1L* | BMI | -0.016(0.005) | 2.28E-03 |
| African American | rs4073401 | *MYT1L* | BMI | -0.016(0.005) | 2.28E-03 |
| African American | rs10518306 | LOC285419 | BMI | -0.026(0.011) | 0.020 |
| African American | rs6062344 | *TCEA2* | BMI | -0.010(0.005) | 0.043 |
| African American | rs6090040 | *TCEA2* | BMI | -0.009(0.005) | 0.047 |
