# Supplementary material for: Genetic Variants Associated with Serum Thyroid Stimulating Hormone (TSH) Levels in European Americans and African Americans from the eMERGE Network
Source: PLoS One. 2014 Dec 1;9(12):e111301. doi: 10.1371/journal.pone.0111301 (PMC4249871; doi:10.1371/journal.pone.0111301)
Supplement: Table S10 — Power calculations for replication/generalization in eMERGE TSH levels study. Power calculations for replication/generalization of SNPs previously associated with serum TSH levels to eMERGE euthyroid European Amercians (EA) and African Americans. SNP rs number, chromosomal location, nearest gene/gene region, coded allele (CA), coded allele frequency (CAF), association summary statistics (betas and p-values), and PubMed ID (PMID) are given for each previously reported association with serum TSH levels in European Americans. Starred (*) CAF represents mean CAF from Taylor et al. Power was calculated for each race/ethnicity using Quanto assuming the previously reported effect size, an additive genetic model, a liberal significance threshold of 0.05, the eMERGE minor allele frequencies, and the eMERGE sample sizes. Power calculations labeled with an asterisk indicate proxy SNPs listed in Table 3 (European Americans) and Table S4 (African Americans) as described in the Methods. (DOCX) [file pone.0111301.s013.docx]

**Table S10: Power calculations for replication/generalization in eMERGE TSH levels study.** Power calculations for replication/generalization of SNPs previously associated with serum TSH levels to eMERGE euthyroid European Amercians (EA) and African Americans. SNP rs number, chromosomal location, nearest gene/gene region, coded allele (CA), coded allele frequency (CAF), association summary statistics (betas and p-values), and PubMed ID (PMID) are given for each previously reported association with serum TSH levels in European Americans. Starred (*) CAF represents mean CAF from Taylor et al. Power was calculated for each race/ethnicity using Quanto assuming the previously reported effect size, an additive genetic model, a liberal significance threshold of 0.05, the eMERGE minor allele frequencies, and the eMERGE sample sizes. Power calculations labeled with an asterisk indicate proxy SNPs listed in Table 3 (European Americans) and Table S4 (African Americans) as described in the Methods.

| **Locus** | | | **Prior Association** | | | | |  |  |  | |
| --- | --- | --- | --- | --- | --- | --- | --- | --- | --- | --- | --- |
| **SNP** | **Chr** | **Gene** | **CA** | **CAF** | **β** | **P-value** | **PMID** | **CAF EA** | **Power EA**  **n=4501** | **CAF AA** | **Power AA**  **n=351** |
| rs10917469 | 1 | *CAPZB* | G | 0.16 | -0.16 | 3.2E-08 | 20826269 | 0.15 | 1.00 | 0.24 | 0.53 |
| rs10917477 | 1 | *CAPZB* | A | 0.51 | -0.06 | 1.54E-08 | 20826269 | 0.48 | 0.74* | 0.49 | 0.14* |
| rs10799824 | 1 | *CAPZB* | A | 0.16 | -0.11 | 3.60E-21 | 23408906 | 0.15 | 0.97* | 0.24 | 0.41 |
| rs334699 | 1 | *NFIA* | A | 0.05 | -0.14 | 5.40E-12 | 23408906 | 0.08 | 0.93* | 0.17 | 0.49 |
| rs13015993 | 2 | *IGFBP5* | A | 0.74 | 0.08 | 3.24E-15 | 23408906 | 0.27 | 0.95 | 0.48 | 0.31 |
| rs10028213 | 4 | *NR3C2* | C | 0.82 | 0.08 | 2.88E-10 | 20826269 | 0.18 | 0.88 | 0.33 | 0.28 |
| rs10032216 | 4 | *NR3C2* | T | 0.78 | 0.09 | 9.28E-16 | 23408906 | 0.19 | 0.95 | 0.42 | 0.37 |
| rs2046045 | 5 | *PDE8B* | T | 0.62 | -0.12 | 2.79E-27 | 22494929 | 0.40 | 1.00 | 0.28 | 0.51 |
| rs6885099 | 5 | *PDE8B* | A | 0.59 | -0.14 | 1.95E-56 | 23408906 | 0.40 | 1.00 | 0.28 | 0.64 |
| rs4704397 | 5 | *PDE8B* | A | 0.40* | 0.21 | 1.64E-10 | 21367965 | 0.39 | 1.00* | 0.28 | 0.92* |
| rs753760 | 6 | *PDE10A* | C | 0.69 | 0.10 | 1.21E-24 | 23408906 | 0.33 | 1.00* | 0.38 | 0.41* |
| rs9472138 | 6 | *VEGFA* | T | 0.29 | -0.08 | 6.72E-16 | 23408906 | 0.28 | 0.96 | 0.19 | 0.21 |
| rs11755845 | 6 | *VEGFA* | T | 0.27 | -0.07 | 1.68E-10 | 23408906 | 0.24 | 0.86 | 0.14 | 0.15 |
| rs9497965 | 6 | *SASH1* | T | 0.42 | 0.05 | 2.25E-08 | 23408906 | 0.30 | 0.41* | 0.18 | 0.08* |
| rs7825175 | 8 | *NRG1* | A | 0.21 | -0.07 | 2.94E-09 | 23408906 | 0.31 | 0.33* | 0.13 | 0.14 |
| rs657152 | 9 | *ABO* | A | 0.34 | 0.06 | 4.11E-10 | 23408906 | 0.38 | 0.84 | 0.43 | 0.19 |
| rs1571583 | 9 | *GLIS3* | A | 0.25 | 0.06 | 2.55E-08 | 23408906 | 0.25 | 0.76 | 0.22 | 0.15 |
| rs17723470 | 11 | *PRDM11* | T | 0.28 | -0.07 | 8.83E-11 | 23408906 | 0.29 | 0.87* | 0.11 | 0.13 |
| rs1537424 | 14 | *MBIP* | T | 0.61 | -0.05 | 1.17E-08 | 23408906 | 0.43 | 0.71 | 0.34 | 0.14 |
| rs11624776 | 14 | *ITPK1* | A | 0.66 | -0.06 | 1.79E-09 | 23408906 | 0.22 | 0.29* | 0.11 | 0.11 |
| rs10519227 | 15 | *FGF7* | A | 0.25 | -0.07 | 1.02E-11 | 23408906 | 0.23 | 0.85 | 0.12 | 0.13 |
| rs17776563 | 15 | *MIR1179* | A | 0.32 | -0.06 | 2.89E-10 | 23408906 | 0.35 | 0.75* | 0.45 | 0.18* |
| rs3813582 | 16 | LOC440389/*MAF* | T | 0.67 | 0.08 | 8.45E-18 | 22494929, 23408906 | 0.31 | 0.97 | 0.25 | 0.25 |
| rs9915657 | 17 | *SOX9* | T | 0.54 | -0.06 | 7.53E-13 | 23408906 | 0.46 | 0.86 | 0.49 | 0.20 |
| rs4804416 | 19 | *INSR* | T | 0.57 | -0.06 | 3.16E-10 | 23408906 | 0.44 | 0.86 | 0.26 | 0.16 |

**References**

1. Panicker V, Wilson SG, Walsh JP, Richards JB, Brown SJ, Beilby JP, Bremner AP, Surdulescu GL, Qweitin E, Gillham-Nasenya I, Soranzo N, Lim EM, Fletcher SJ, Spector TD (2010) A locus on chromosome 1p36 is associated with thyrotropin and thyroid function as identified by genome-wide association study. Am J Hum Genet 87: 430-435. S0002-9297(10)00418-0 [pii];10.1016/j.ajhg.2010.08.005 [doi].

2. Rawal R, Teumer A, Volzke H, Wallaschofski H, Ittermann T, Asvold BO, Bjoro T, Greiser KH, Tiller D, Werdan K, Meyer Zu Schwabedissen HE, Doering A, Illig T, Gieger C, Meisinger C, Homuth G (2012) Meta-analysis of two genome-wide association studies identifies four genetic loci associated with thyroid function. Hum Mol Genet 21: 3275-3282. dds136 [pii];10.1093/hmg/dds136 [doi].

3. Medici M, van der Deure WM, Verbiest M, Vermeulen SH, Hansen PS, Kiemeney LA, Hermus AR, Breteler MM, Hofman A, Hegedus L, Kyvik KO, den HM, Uitterlinden AG, Visser TJ, Peeters RP (2011) A large-scale association analysis of 68 thyroid hormone pathway genes with serum TSH and FT4 levels. Eur J Endocrinol 164: 781-788. EJE-10-1130 [pii];10.1530/EJE-10-1130 [doi].
